# Functional and antigenic constraints on the Nipah virus fusion protein

**DOI:** 10.1073/pnas.2529505123

**Published:** 2026-02-06

**Authors:** Brendan B. Larsen, Sheri Harari, Risako Gen, Cameron Stewart, David Veesler, Jesse D. Bloom

**Affiliations:** ^a^Basic Sciences Division and Computational Biology Program, Fred Hutchinson Cancer Center, Seattle, WA 98109; ^b^Department of Biochemistry, University of Washington, Seattle, WA 98195; ^c^HHMI, Seattle, WA 98195

**Keywords:** class I fusion protein, deep mutational scanning, pseudovirus, paramyxovirus

## Abstract

Nipah virus is a highly pathogenic virus in the family *Paramyxoviridae* that utilizes two distinct surface glycoproteins to infect cells. The receptor-binding protein (RBP) binds host receptors whereas the fusion protein (F) merges viral and host membranes. Here, we use nonreplicative pseudoviruses to safely measure the effects of all F single amino acid residue mutations on its cell entry function and neutralization by monoclonal antibodies. We compare mutational tolerance in F with previous experimental measurements for RBP and show that F is much more functionally constrained than the RBP. We also identify mutationally intolerant sites on the F trimer surface and core that are critical for proper function, and describe mutations that are candidates for stabilizing F in the prefusion conformation for vaccine design. We quantify how F mutations affect neutralization by six monoclonal antibodies, and show that the magnitude of mutational effects on neutralization varies among antibodies. Our measurements of mutational effects on Nipah virus F predict the ability of the antibodies to neutralize the related Hendra virus. Overall, our work defines the functional and antigenic constraints on the F protein from an important zoonotic virus.

## Significance

Nipah virus sporadically spills over into humans, where it is often fatal. The Nipah fusion (F) protein is necessary for infection, and is a target for vaccines and antibody therapies. To better understand the constraints on this protein, we experimentally measured how ~8,500 single amino acid mutations to F affected its function using pseudoviruses that enable the safe study of protein mutants without the generation of actual replicative virus. We examined the effects of these mutations in the context of structural data and publicly available Nipah virus sequences to characterize the constraints that shape F protein evolution. This work has implications for understanding paramyxovirus fusion proteins, and informs the development of vaccines and monoclonal antibody therapies.

Nipah virus is a bat-borne paramyxovirus that recurrently spills over to humans, causing severe disease with case fatality rates of 40 to 90% (1–3). Since the first described outbreak in Malaysia in 1998, sporadic spillovers have occurred in India and Bangladesh, with Bangladesh reporting near-annual cases since 2001 (2, 4–6). While these infections typically remain isolated, limited human-to-human transmission has been reported, raising the possibility of wider outbreaks (6, 7).

Nipah virus, like other viruses in the family *Paramyxoviridae*, infects cells through the coordinated action of two different surface glycoproteins: the tetrameric receptor-binding protein (RBP, also known as G) and the trimeric fusion protein (F). F is a class I fusion protein that is cleaved by cathepsins in the endosomal compartment, through a recycling mechanism, to activate it prior to its incorporation into virions (8–11). Once the RBP binds to the host receptors ephrin-B2 or ephrin-B3 (EFNB2/3), it undergoes a conformational change which triggers F to irreversibly transition from its metastable prefusion conformation to an extended postfusion conformation (12–15). During this process, F undergoes large-scale structural rearrangements and inserts an exposed fusion peptide into the host cell membrane, ultimately forming a highly stable six-helix bundle in postfusion F (16–18).

Paramyxovirus RBP tetrameric structures differ substantially, reflecting the wide variety of host receptors used for cell entry (19–23). In contrast, paramyxovirus F proteins show high structural conservation to each other in the pre- and in the postfusion conformations, suggesting shared mechanisms of F function and triggering mechanisms across divergent viruses (17, 24, 25).

Understanding the precise molecular interactions between RBP and F during fusion initiation remains a critical knowledge gap for Nipah virus and other paramyxoviruses. Recently, cryo-EM captured a RBP/F complex from a related paramyxovirus, human parainfluenza virus, which showed one of the RBP heads rests on top of F and likely inhibits the triggering of F until it is released via receptor binding (26). However, there is evidence that the mechanism of F-triggering likely differs among the various paramyxovirus genera (17, 27, 28). For Nipah virus and other members of the genus *Henipavirus*, the precise interaction between RBP and F remains unknown.

Despite the fact that Nipah virus causes severe disease in humans, there are currently no approved vaccines or therapeutics for this virus. Vaccines based on prefusion-stabilized F elicit neutralizing antibodies in mice whereas vaccines based on postfusion F do not (29, 30). Monoclonal antibodies targeting F have shown exceptional promise in preventing disease in animal models, outperforming a best-in-class RBP-directed antibody (31–34). However, evolution can rapidly erode antibody effectiveness for other viruses, sometimes through single mutations which abrogate antibody binding to the viral glycoproteins (35, 36). A better understanding of how mutations affect antibody neutralization can help guide the development of monoclonal antibodies and antibody cocktails with optimized resilience to viral evolution (37).

To provide an in-depth characterization of the function and antigenicity of the Nipah virus F protein, we performed deep mutational scanning on the Nipah F ectodomain using a pseudotyped lentiviral platform that enables us to safely measure the functional and antigenic impact of F mutations without using an authentic Nipah virus isolate. We experimentally measured the effects of all possible single amino acid residue mutations to Nipah virus F on cell entry and neutralization mediated by six monoclonal antibodies. These data provide detailed information about the functional role of different regions of F, help identify candidate stabilizing mutations for vaccines, and quantify the resilience of different antibodies to possible future viral evolution.

## Results

### Structure and Evolution of Nipah virus F.

**Fig. 1.**
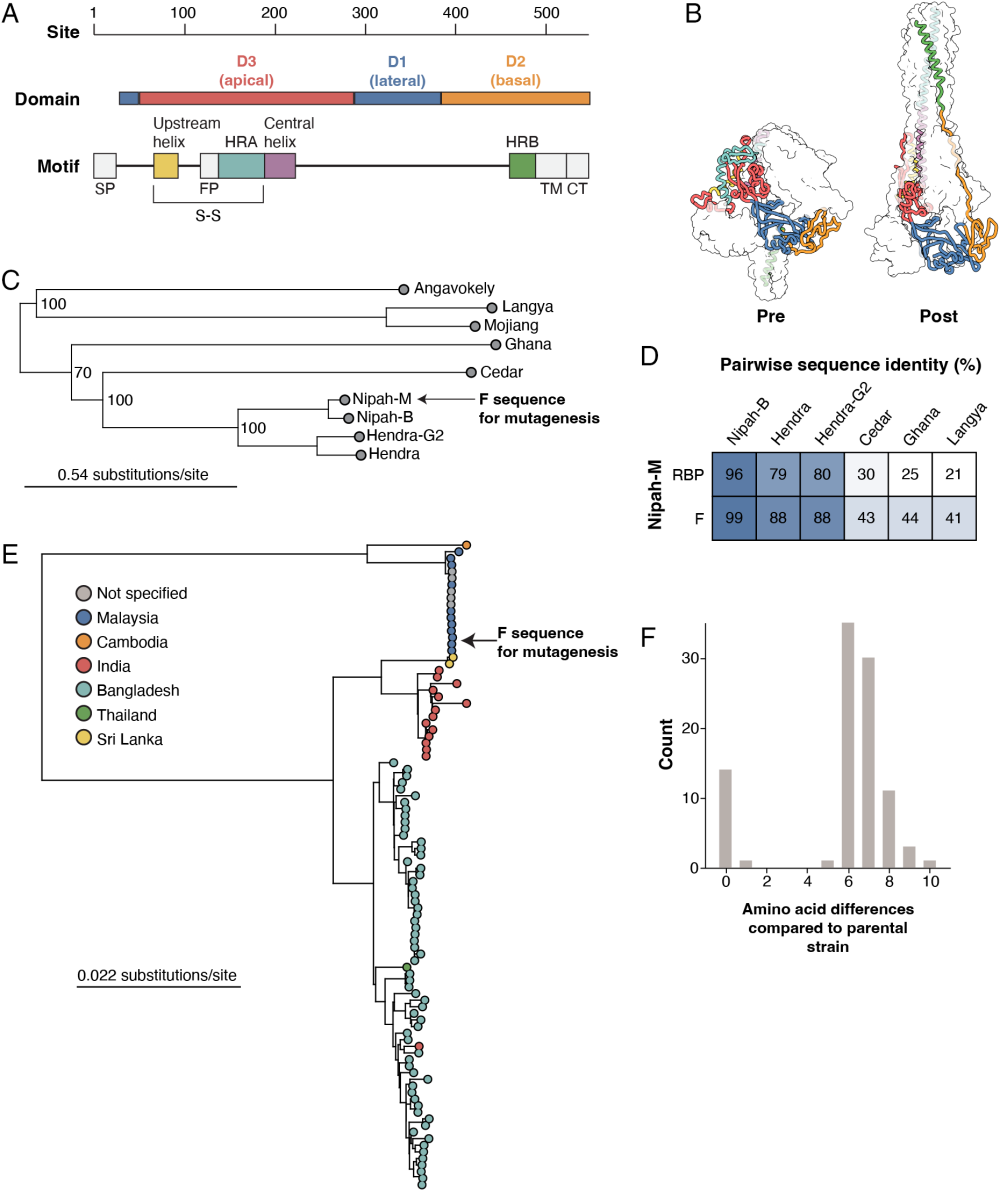
Functional domains and evolution of Nipah F. (*A*) Major structural domains and motifs in Nipah F. The motifs are abbreviated as follows. Signal Peptide (SP), Heptad-repeats A and B (HRA,HRB), Fusion peptide (FP), transmembrane region (TM), and cytoplasmic tail (CT). The disulfide bond between F1 and F2 is indicated with an S–S. (*B*) Nipah F trimer with one monomer colored by domain or motif as in panel A, and the other two monomers shown as transparent white surfaces. The prefusion structure is based on PDB 5EVM, the postfusion structure is an AlphaFold2-generated structure of postfusion Nipah virus F using the postfusion Langya virus F as template (PDB 8TVE) (21). (*C*) Maximum-likelihood phylogenetic tree built from representative publicly available henipavirus whole-genome sequences. Arrow points to Nipah Malaysia, the parental sequence used in the deep mutational scanning experiments. (*D*) Pairwise amino acid sequence identity between Nipah Malaysia RBP and F with other representative henipaviruses. (*E*) Maximum-likelihood phylogenetic tree of all Nipah virus whole-genome sequences deposited in GenBank. Circles are colored by the country of origin provided in GenBank records. The arrow points to the Nipah Malaysia strain used as the parent for the deep mutational scanning experiments. (*F*) Amino acid differences in full-length F sequences between the Nipah Malaysia parental strain used in our study and all other Nipah F sequences.

Nipah virus F is a trimeric, type I transmembrane protein made up of three major domains named D1, D2, and D3, which correspond to the lateral, basal, and apical faces, respectively (Fig. 1*A*). In the prefusion structure, heptad repeat A (HRA) forms a series of alpha-helices and beta-sheets which transition into a single long alpha-helix together with the central helix in the postfusion structure (Fig. 1*B*). Heptad repeat B (HRB) forms a three-helix coil leading to the viral membrane in the prefusion F conformation, which refolds to interact with the periphery of the postfusion HRA coiled-coil, leading to the aforementioned six-helix bundle (Fig. 1*B*). An upstream helix is proximal to the central helix, with which it forms a disulfide bond, and that has been shown to have an important role in F triggering (38, 39).

Nipah virus is a member of the *Henipavirus* genus, which includes other viruses that cause disease in humans, such as Hendra virus and the recently described Langya virus (Fig. 1*C*). Across these paramyxoviruses, F is more conserved than RBP at the amino acid level (Fig. 1*D*). For the parental Nipah virus F amino acid sequence for our deep mutational scan, we used a codon-optimized F gene from the reference strain from GenBank (NC_002728.1), which was sequenced during the first outbreak in 1999 in Malaysia (Fig. 1*E*). Among all publicly available sequences, there is a maximum of ten amino acid differences between the strain used in deep mutational scanning and all other Nipah virus F sequences, despite broad geographical separation and 25 years of evolution (Fig. 1*F*). These data are suggestive of significant functional constraints on Nipah virus F.

### Pseudovirus Deep Mutational Scanning Libraries of Nipah Virus F.

We performed pseudovirus deep mutational scanning using a previously described approach based on pseudotyped lentiviral particles (*SI Appendix*, Fig. S1) (40, 41). These pseudoviruses encode no viral proteins other than F and can only undergo a single round of cell entry. They are therefore not fully infectious agents as they do not replicate or cause disease, making them a safe tool to study proteins from deadly pathogens such as Nipah virus. For our experiments, we truncated portions of the cytoplasmic tail of F and RBP, as this increased pseudovirus titers (see previous work (42, 43), *Materials and Methods* and *SI Appendix*, Fig. S2).

To generate the F mutant libraries, we used 300 nucleotide oligo pools (oPools) synthesized by Twist Biosciences that contained all possible amino acid mutations to the ectodomain of F (site 29 to 481) and assembled them into lentiviral vectors using Golden Gate Assembly (*SI Appendix*, Fig. S3), an approach loosely based on previous work (44–46). We added barcodes consisting of 16 random nucleotides downstream from the stop codon of each F variant; these barcodes are subsequently linked to the full F sequence by long-read PacBio sequencing which then facilitates downstream sequencing by enabling each variant to be identified by Illumina sequencing of its barcode (40).

We made two replicate libraries (LibA and LibB), which have unique mutation-barcode linkages, followed by generation of cell-stored lentiviral libraries in 293 T-rtTA cells, where each cell contains a single provirus with a unique Nipah F variant (40, 41). There were 56,368 and 62,614 unique barcoded variants for LibA and LibB, respectively. 69% of these variants contained a single amino acid mutation relative to the parental sequence, with the remainder being unmutated (~11%) or variants with >1 mutation (~20%) (*SI Appendix*, Fig. S4*A*). Most mutations relative to the parental sequence matched the exact codon sequence used for the oPool design (*SI Appendix*, Fig. S4 *B* and *C*). Most of the variants with >1 mutation likely arose from lentiviral recombination (47, 48), an unavoidable step in generating our pseudovirus libraries (*SI Appendix*, Fig. S4*D*). These results demonstrate low error rates in the oPool synthesis and library assembly.

### Measuring Effects of Nipah F Mutations on Cell Entry.

To measure the effects of F mutations on cell entry, we used an approach similar to that recently described in our deep mutational scanning of Nipah RBP (41). Specifically, we quantified the ability of pseudoviruses with each F protein mutant to enter a cell line expressing a Nipah virus receptor protein relative to control pseudoviruses that use a different viral entry protein (VSV-G) to enter cells. In these experiments, mutations to F can affect cell entry through several different processes, including by affecting protein expression, folding, stability, and triggering by RBP. Mutation effects are reported as log_2_ transformed values, where −1 represents a two-fold reduction in cell entry relative to the parental unmutated F protein. Since ~20% of the variants contain multiple mutations (*SI Appendix*, Fig. S4*A*), we fit global epistasis models to estimate the effects of single mutations from the entire library of single and multiple mutant variants (49). The effects of mutations determined from the global epistasis model fitting were highly correlated with the effects of mutations in the single-mutant variants only (*Materials and Methods* and *SI Appendix*, Fig. S5).

Nipah virus can use two different receptors, ephrin-B2 or -B3 (EFNB2/3), to enter cells (15, 50, 51). We previously generated CHO cells that stably express either *Pteropus alecto* bat ephrin-B2 or -B3 (CHO-bEFNB2/3) (41). The use of cells expressing bat rather than human ephrin receptors reduces the possibility that our experiments could uncover potentially hazardous information about F protein mutations that might adapt the virus to better infect human cells (41, 52, 53). In prior work, we found that the effects of mutations on the cell entry function of Nipah RBP differ somewhat between cells expressing bEFNB2 versus bEFNB3 (41). Although F is not directly involved in receptor binding, prior work has suggested that F is triggered more effectively by RBP binding to EFNB2 relative to EFNB3, possibly because RBP binds to EFNB2 more strongly (54). However, we found the average effects of F mutations at each site on cell entry were highly correlated between measurements made on bEFNB2 versus bEFNB3 expressing cells, in sharp contrast to similar measurements for RBP mutations (r = 0.99 for F versus 0.82 for RBP; *SI Appendix*, Fig. S6). Therefore, at least in our experimental system, the effects of F mutations on cell entry are largely independent of whether the target cells express bEFNB2 or bEFNB3, a result that makes sense in light of F’s primary function being membrane fusion rather than receptor binding. For all subsequent results in this paper, we report the effects of mutations from experiments performed in CHO-bEFNB3 cells.

In total, we reliably measured the effects of 8,449 F mutations on cell entry, which represents 98.2% of all possible amino acid mutations. Each mutation had an average of 5.9 and 6.2 associated unique barcodes for LibA and LibB, respectively (*SI Appendix*, Fig. S7*A*). The effects of mutations on entry were highly correlated between our replicate variant libraries (r = 0.94; *SI Appendix*, Fig. S7*B*), and throughout the rest of this paper we report the average of the measured effects across libraries.

### Effects of Nipah F Mutations on Cell Entry.

**Fig. 2.**
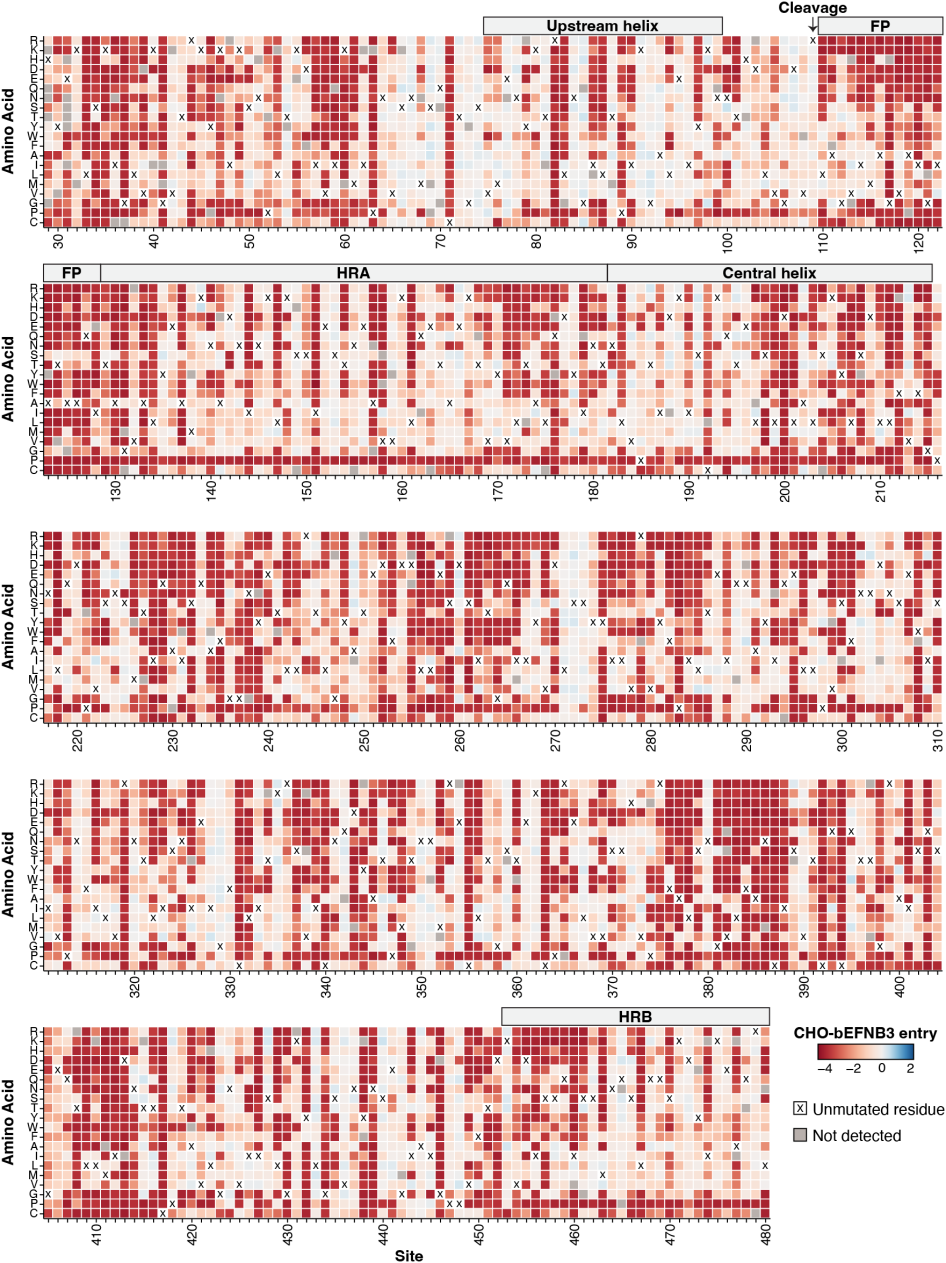
Effects of Nipah F mutations on entry in CHO-bEFNB3 cells. Each mutation is colored by its effect on cell entry. Effects on cell entry range from highly deleterious (red) to neutral (white) to slightly beneficial (blue). The amino acid identity at each site in the parental F from the Malaysia strain is indicated with a black “X.” Mutations that were missing in our library and so lack a measurement are colored gray. The experiments were performed in CHO cells that stably express ephrin-B3 from the bat species *Pteropus alecto*. See https://dms-vep.org/Nipah_Malaysia_F_DMS/visualizations/posts/CellEntryHeatmapWrappedZoomable.html for an interactive version of the plot.

**Fig. 3.**
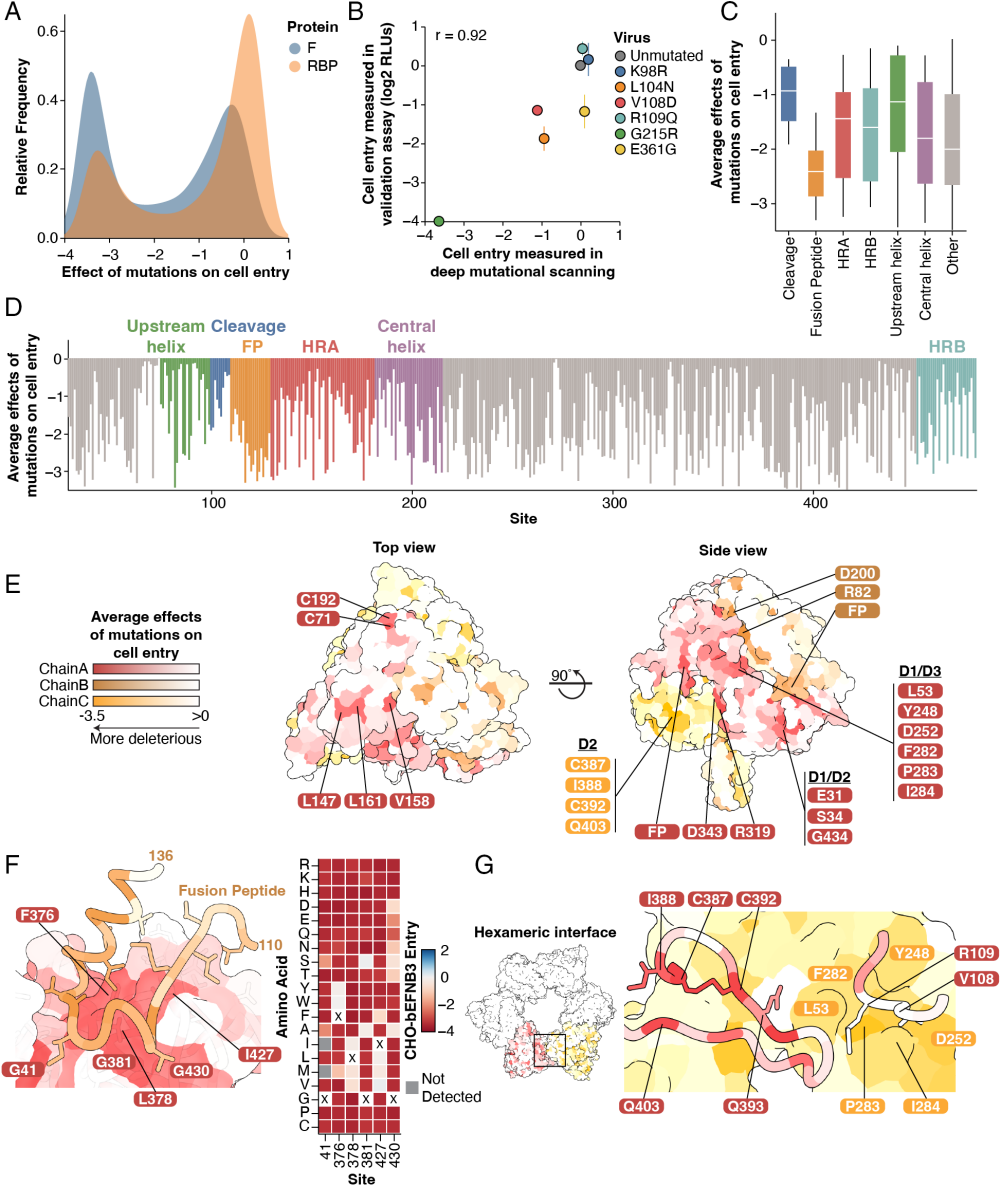
Effects of F mutations on cell entry in context of the protein structure. (*A*) Distribution of the effects of all amino acid mutations to F or RBP on cell entry in CHO-bEFNB3 cells, with the measurements for F from the current study and RBP from Larsen et al. (41). More negative values indicate mutations more strongly impair cell entry. (*B*) Validation of cell entry effects in CHO-bEFNB3 cells. Correlation between deep mutational scanning cell entry measurements and individual pseudoviruses expressing the respective mutation. Individual mutations were chosen to span a range of entry effects. For validation assays, relative light units (RLUs) were measured by a luciferase assay and compared to the unmutated values. Measurements were done in triplicate. (*C*) Min-max boxplot showing the average effects of all amino acid mutations at each site for different regions of F. (*D*) Average effect of mutations at each site across the F ectodomain, colored by protein domain or region. (*E*) Average effects of mutations at each site mapped onto the prefusion F structure (PDB: 5EVM), viewed from the *Top* and *Side*. Highly constrained, surface-exposed residues are labeled. Each protomer is colored with a separate color. (*F*) The fusion peptide rests on a highly constrained surface of the nearby protomer in the prefusion F structure. Sites are colored by the average effects of mutations on cell entry. Key constrained interface residues are labeled. The heatmap (*Right*) shows the effects of all mutations at the sites labeled on chain A on the structure. (*G*) Nipah virus F trimers are arranged in a hexameric ring, as observed in the crystal lattice described in PDB: 5EVM. The boxed area at the interface of the trimers is shown in more detail on the *Right*. Trimers are colored separately. Specific residues at the interface are labeled.

Examination of our comprehensive measurements of mutational effects on cell entry shows that the F protein is highly mutationally constrained (Fig. 2), which is consistent with the low rate of protein evolution observed in circulating sequences (Fig. 1 *D* and *F*). We compared the effects of mutations on entry with our previously generated RBP data (41). In general, F mutations were much more deleterious for cell entry compared to RBP mutations (Fig. 3*A*). This result indicates the lower rate of natural sequence evolution in F relative to RBP is a result of higher functional constraints on the protein.

To validate our deep mutational scanning measurements of the effects of F mutations on cell entry, we generated pseudoviruses expressing different individual F mutations selected to span a range of effects, and compared their entry relative to the unmutated parental strain. Entry into cells for the individual pseudovirus mutants was highly correlated with the deep mutational scanning measurements of the effects of these mutations (r = 0.92; Fig. 3*B*).

Based on the average effects of F mutations at each site on cell entry, the fusion peptide is the most constrained region, while the region upstream of the cleavage site is fairly tolerant to mutations (Fig. 3 *C* and *D*). These data are congruent with previous low-throughput mutagenesis experiments on Nipah F within the cleavage and fusion peptide regions (10, 55–57). Of the four helical regions, the upstream helix was the least constrained (Fig. 3*C*).

To understand the structural context of mutational constraint, we mapped the average effects of mutations at each site onto the trimeric prefusion structure (Fig. 3*E*). The surface-exposed apex of the protein is largely tolerant to mutations, with the exception of two cysteine residues which form a disulfide bond between F1 and F2 (C71/C192) and three hydrophobic residues (L147, V158, and L161; Fig. 3*E*).

The surface-exposed lateral face of the trimer is more constrained than the apex. Some of the most highly constrained surface-exposed sites are charged residues, including R82, D200, R319, and D343 (Fig. 3*E*). There are five additional patches of surface-exposed residues on the lateral faces that are highly constrained. These include the fusion peptide interface between the monomers, a region consisting exclusively of residues in D2 (C387, I388, C392, Q403), a region of D1/D2 (E31, S34 G434), and a region in D1/D3 (L53, Y248, D252, F282, P283, I284) (Fig. 3*E*).

The fusion peptide (sites 110 to 128) is inserted into a groove in the opposing protomer (Fig. 3*F*). Fusion peptide residues directly in this interface were more constrained than those outside of it (Fig. 3*F*). The cavity-forming residues surrounding the fusion peptide are primarily composed of hydrophobic and glycine residues, and the effects of mutations at these sites are defined by specific amino acid biochemical properties. For example, at sites G41 and G381, mutations to amino acids with small side chains (A,S) were less deleterious than other mutations (Fig. 3*F*). These data suggest that efficient sequestration of the fusion peptide is critical for proper F function.

Prior work has suggested that Nipah virus F trimers may assemble into hexameric rings (25, 58). Two of the surface-exposed patches of constrained residues (D2 and D1/D3; Fig. 3*G*) are within the interface between trimers of the hexamer (Fig. 3*G*). In one trimer, the loop containing the cleavage site at R109 contacts the opposing trimer at sites in the highly constrained D1/D3 patch, including L53, P283, and I284. The constrained patch from D2, including C387, I388, C392, and Q403 also contact the opposing trimer (Fig. 3*G*). While the cysteine residues are important for maintaining overall protein structure, these data are consistent with an important functional role of the hexameric interfaces in modulating fusion (58).

### Effects of Mutations Mapped to the Postfusion Structure.

**Fig. 4.**
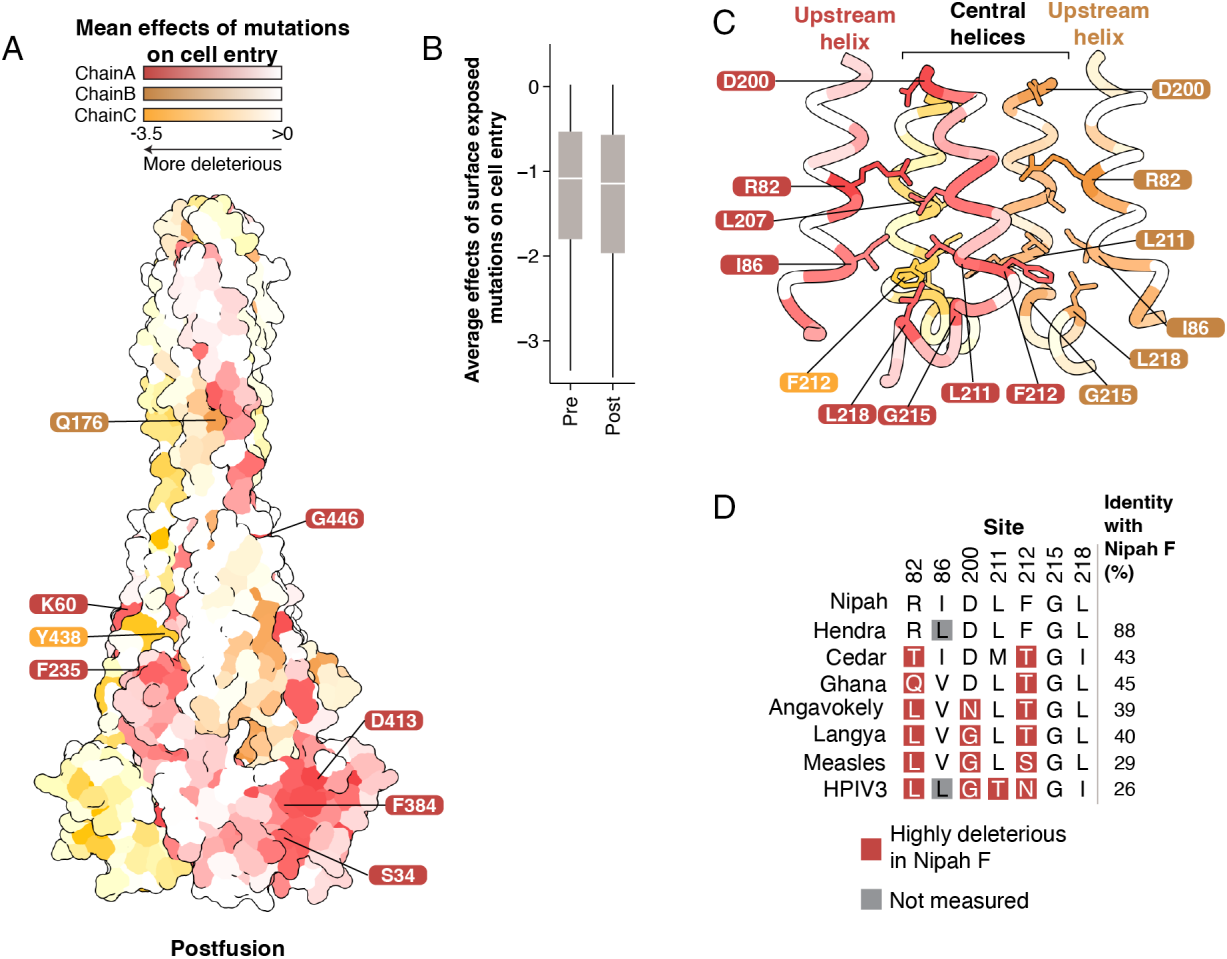
Effects of mutations on cell entry mapped on the postfusion conformation of the F protein. (*A*) Average effects of mutations at each site on cell entry mapped onto an AlphaFold2-generated structure of postfusion Nipah virus F using the postfusion Langya virus F as template (PDB 8TVE) (21). Highly constrained surface-exposed sites that were buried in the prefusion structure are labeled. (*B*) Effects of mutations at all surface-exposed sites in the pre- and postfusion conformations. Boxed regions show the mean, Q1, and Q3. (*C*) Average effects of mutations at each site mapped onto a region near the base of the central helix in the postfusion conformation. R82 in the upstream helix is pointed inward toward the central helices. F212 is buried in a hydrophobic patch formed by I86, L211, G215, and L218. (*D*) Amino acid identity of representative paramyxoviruses at constrained sites labeled in (*B*). Mutations at sites R82, D200, L211, and F212 that are highly deleterious for cell entry of Nipah F are present as the wildtype amino acid in F proteins of some more divergent paramyxoviruses. Overall pairwise amino acid identity to Nipah F is shown on the *Right*.

Since Nipah virus F transitions from a prefusion to a postfusion conformation during viral entry, we next examined the effects of mutations in the postfusion structure. Some highly constrained residues that were buried in the prefusion structure have moved to the surface (Fig. 4*A*), but the average effects of surface exposed residues remains similar to the prefusion structure (Fig. 4*B*).

One of the major structural rearrangements is located in D3 and is associated with the extension of HRA into a single helix continuous with the central helix, and the packing of the three HRA helices as a coiled-coil in the center of the trimer (59). To identify key residues involved in this transition, we examined the base of the central helix and the associated upstream helix in the postfusion structure. This region contains many sites that are highly constrained, including R82, D200, F212, and G215 (Fig. 4*C*). R82 and D200 are the most mutationally intolerant amino acids in the upstream and central helices, respectively (*SI Appendix*, Fig. S8), and in the prefusion structure they are partially surface-exposed (Fig. 3*E*). However, in the postfusion structure, both R82 and D200 are buried, near the core of the trimer (Fig. 4*C*). F212 is buried in a hydrophobic pocket that includes sites I86, L211, and L218 (Fig. 4*C*). Despite the high constraint observed at these sites for Nipah virus F, the F proteins of other highly divergent paramyxoviruses with <50% amino acid identity with Nipah F contain amino acids at sites 82, 200, 211, and 212 that are highly deleterious for cell entry in Nipah virus F (Fig. 4*D*). This finding suggests the effects of specific F mutations on cell entry can differ in highly divergent paramyxoviruses, likely due to epistasis with other changes that alter sequence-function constraints.

### Identification of Candidate Mutations for Stabilizing F in the Prefusion Conformation.

For many viral vaccines, adding mutations to the fusion protein that stabilize its prefusion conformation can increase the vaccine-elicited neutralizing antibody titers (60, 61). Several prior studies have described mutations that stabilize Nipah or Hendra F in the prefusion conformation (21, 29, 30, 62), but the identification of additional stabilizing mutations could further improve immunogen design. One previously described Nipah virus F stabilized construct consisted of two cysteine mutations to form an additional disulfide bond between F1 and F2 (L104C/I114C), a space-filling mutation (L172F), and a proline mutation in the central helix (S191P) (29).

**Fig. 5.**
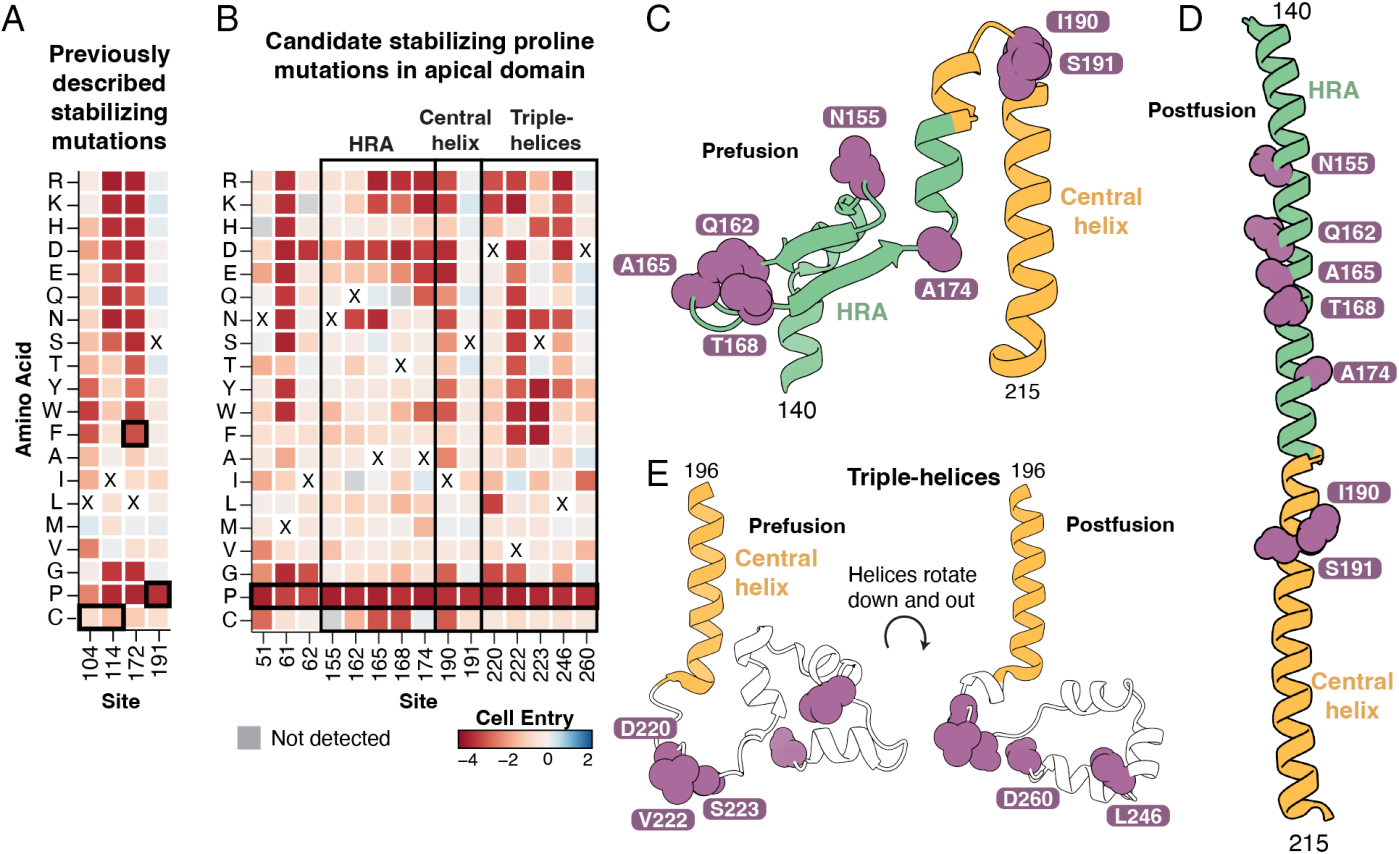
Candidate prefusion stabilizing mutations. (*A*) Effects of single mutations on cell entry at sites with previously described (29) prefusion stabilizing mutations (L104C/I114C, L172F, and S191P; each surrounded by a black box). The two cysteine mutations create an additional disulfide bond between F1 and F2, L172F mutation is space-filling, and S191P adds a proline that disrupts helical formation in the postfusion conformation. These mutations are likely deleterious for cell entry even though they increase prefusion F stability because they restrict conformational transitions necessary for F-mediated fusion. (*B*) Effects of single mutations on cell entry at sites in the apical domain where we suggest that proline mutations might increase the stability of prefusion F. Sites with highly deleterious proline mutations, and at least some tolerated mutations, likely disrupt helical formation or conformational flexibility, restricting the transition to the postfusion conformation. Heavy black box is around the candidate proline mutations, the light black box surrounds different regions of the apical domain. (*C*) Sites with candidate stabilizing mutations (purple) mapped onto HRA (green) and the central helix (yellow) from the prefusion structure. (*D*) Sites with candidate stabilizing mutations mapped onto the HRA and central helix from postfusion structure. (*E*) Sites with candidate stabilizing mutations in the triple-helix region downstream from the base of the central helix mapped onto the pre- and postfusion structures.

The transition of F to the postfusion conformation is necessary for it to mediate cell entry, so we predict prefusion-stabilizing F mutations should be deleterious for cell entry in our deep mutational scanning. As expected, the two previously described mutations that individually stabilize prefusion F (L172F and S191P) were deleterious for cell entry in our measurements (Fig. 5*A*). Two previously described cysteine mutations (L104C and I114C) that stabilize prefusion F by forming a disulfide bond were relatively well tolerated for cell entry in our measurements (Fig. 5*A*), likely because we only measured the impact of single mutations and both cysteine mutations must be made simultaneously to form the stabilizing disulfide bond.

To identify additional candidate prefusion stabilizing mutations to Nipah virus F, we searched for sites where mutations to prolines were highly deleterious, but mutations to most other amino acids were well tolerated, similar to the known stabilizing mutation S191P (Fig. 5*A*). Prolines are uniquely unfavorable in the helical conformations that are adopted in the postfusion conformation, so sites that tolerate mutations to other amino acids but not proline will often be ones where folding of the prefusion conformation is tolerant of mutations but proline specifically impairs cell entry by preventing the transition to the postfusion conformation. We focused on sites in the apical domain since it undergoes a large conformational shift that results in a long alpha-helix in the postfusion conformation (59), and excluded sites that were in helices or strands in the prefusion conformation because proline mutations in these secondary-structure elements are likely to cause misfolding of the prefusion conformation. We identified 15 sites where prolines were highly deleterious but at least four other mutations were tolerated (Fig. 5*B*); five of these sites were in HRA and two were in the central helix (Fig. 5 *C* and *D*), while five were in the triple-helix region just downstream from the central helix base (Fig. 5*E*). Overall, these mutations represent promising candidates for further investigation as prefusion-stabilizing F mutations for vaccine design, although further experimental characterization beyond the scope of the current study would be needed to confirm which of these mutations actually effectively stabilize prefusion F and retain native antigenicity.

### Effects on Cell Entry of Mutations Found in Circulating Nipah and Hendra Viruses.

We collated all mutations in the F protein from publicly available Nipah virus sequences and examined their effects on cell entry as measured in our experiments. Nearly all naturally occurring Nipah virus mutations in the regions we mutagenized (the F ectodomain) were neutral (*SI Appendix*, Fig. S9). We then took the same approach with available Hendra virus F sequences, which share ~88% amino acid identity with Nipah F. If epistasis with other mutations is modifying the effects of mutations in Hendra virus F versus Nipah virus F, we would expect some naturally occurring Hendra virus mutations to be deleterious for cell entry in Nipah virus F. Instead, nearly all mutations in Hendra virus F relative to the Nipah virus F parental strain were also neutral for cell entry (*SI Appendix*, Fig. S9).

### Measurement of How All Mutations Affect Neutralization Quantifies Antibodies Resilience to F-Protein Mutations.

We measured how all functionally tolerated mutations to Nipah virus F affect neutralization by six monoclonal antibodies that target the apical (12B2, 2D3, 4H3), lateral (1A9, 1F2), and basal (2B12) faces (33, 63). To measure how F mutations affect antibody neutralization, we performed selections on our replicate pseudovirus libraries with varying concentrations of antibody as previously described (40, 41). Briefly, we quantify infection by pseudoviruses with each F protein mutant relative to a standard that is unaffected by anti-F antibodies in order to measure the impact of each mutation on neutralization.

**Fig. 6.**
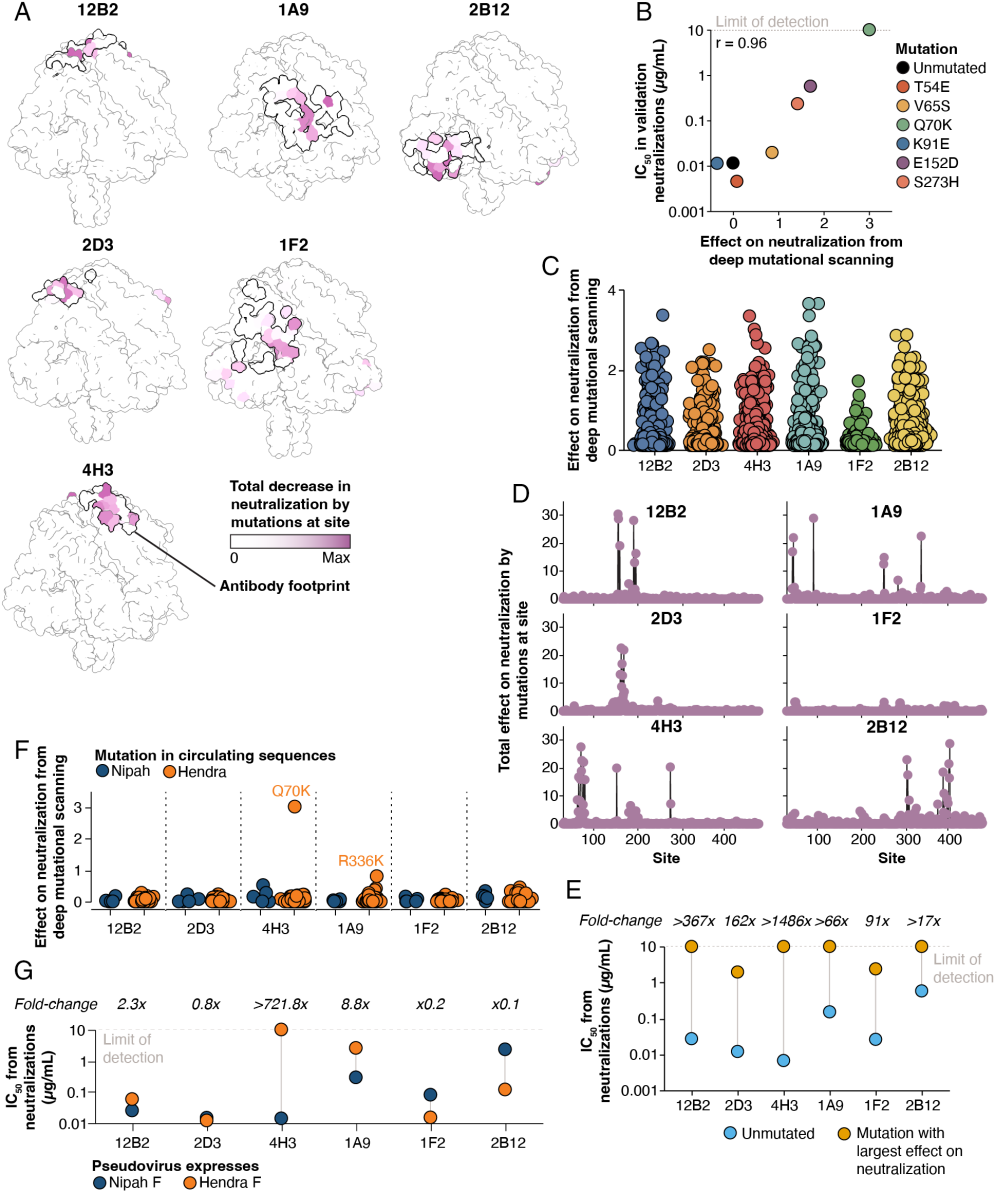
Effects of single mutations to F on antibody neutralization. (*A*) Prefusion Nipah F colored by the total effect of mutations at each site on neutralization by each antibody. The thick black line shows the binding footprint of each antibody from the experimentally determined structures (see *Materials and Methods* for PDB accession numbers). Coloring is scaled to the maximum value separately for each antibody. See https://dms-vep.org/Nipah_Malaysia_F_DMS/visualizations/posts/AntibodyEscapeHeatmaps.html for links to interactive heatmaps that show the effects of all mutations on neutralization for each antibody. (*B*) Correlation of mutation effects on neutralization by 4H3 between deep mutational scanning and traditional pseudovirus neutralization assays with the indicated F mutant. Individual mutations were selected to span a range of effects on neutralization. (*C*) Extent of decreases in neutralization from each antibody caused by all mutations as measured in the deep mutational scanning. Each point is the effect of a different mutation. (*D*) Total decrease in neutralization by all mutations at each site. (*E*) Comparison of IC_50_ values measured in traditional neutralization assays between pseudoviruses expressing unmutated F or F with the single mutation with the greatest effect on neutralization for that antibody in the deep mutational scanning. (*F*) Measured effects of all unique mutations observed in publicly available natural Nipah and Hendra F sequences on antibody neutralization in the context of Nipah F. Each point is a different amino acid mutation observed at least twice in a Nipah or Hendra F sequence. (*G*) Differences in neutralizing potency between pseudoviruses bearing either Nipah or Hendra F.

The mutations that affected neutralization by the antibodies were almost entirely surface exposed and directly mapped to the structurally determined epitopes of each antibody (Fig. 6*A*). To validate the ability of deep mutational scanning to accurately predict neutralization measurements, we generated pseudoviruses expressing different F mutations spanning a range of effects, and measured their neutralization with antibody 4H3. The IC_50_ estimates against the different F mutants as measured in these pseudovirus neutralization assays were highly correlated with the deep mutational scanning data (r = 0.96, Fig. 6*B* and *SI Appendix*, Fig. S11*A*).

We identified mutations that affected neutralization by all antibodies, but the number and extent of the effects varied among antibodies (Fig. 6 *C* and *D*). Fewer mutations affected neutralization mediated by antibody 1F2 relative to other antibodies tested: 1F2 neutralization was also affected by only a few specific amino acid changes, primarily at sites 51 and 286, while the other antibodies were affected by a much broader range of amino acid changes at multiple sites (Fig. 6 *C* and *D* and *SI Appendix*, Fig. S10). To confirm mutations had smaller effects on 1F2 neutralization than the other antibodies, we generated individual pseudoviruses containing the mutation identified in the deep mutational scanning data as most affecting neutralization by each antibody, and tested these pseudoviruses in traditional neutralization assays (Fig. 6*E* and *SI Appendix*, Fig. S11*B*). While single mutations completely escaped neutralization mediated by four of the antibodies, two antibodies (1F2 and 2D3) retained detectable neutralization even against the F protein carrying the mutation that most impacted neutralization by that antibody (Fig. 6*E*).

We examined whether mutations measured in the deep mutational scanning to reduce neutralization by any of the antibodies are found in naturally occurring Nipah F sequences available in GenBank. This analysis revealed that no mutations present in circulating Nipah F sequences have strong effects on neutralization by any of the antibodies (Fig. 6*F*).

### Deep Mutational Scanning Measurements of How Mutations to Nipah F Affect Antibody Neutralization Can Predict Which Antibodies Neutralize Hendra F.

To determine if our measurements of how mutations affect neutralization of Nipah virus F generalize to Hendra virus, a closely related henipavirus, we examined whether any mutations in Hendra virus F relative to Nipah virus F were measured to reduce antibody neutralization in the deep mutational scanning. One mutation found in all Hendra virus sequences (Q70K) strongly escaped antibody 4H3 neutralization in our Nipah F deep mutational scanning data, and another mutation found in all Hendra virus sequences (R336K) moderately reduced neutralization by antibody 1A9 (Fig. 6*F*). Therefore, if the deep mutational scanning measurements can be generalized from Nipah virus F to Hendra virus F, we would predict that 4H3 does not neutralize Hendra and 1A9 has modestly reduced neutralization, but the other antibodies should still neutralize Hendra virus well. To test this prediction, we generated pseudoviruses expressing Nipah virus RBP and either Nipah virus or Hendra virus F and compared their neutralization in a traditional luciferase-based assay (Fig. 6*G* and *SI Appendix*, Fig. S11*C*). 4H3 did not neutralize pseudoviruses containing Hendra F even at concentrations up to 10 µg/mL, while 1A9 neutralized Hendra virus F-harboring pseudovirus with an 8.8-fold higher IC_50_ relative to Nipah virus F (Fig. 6*G*). The remaining antibodies neutralized pseudoviruses expressing either Hendra or Nipah virus F at mostly similar levels, although some antibodies had mildly reduced neutralization with Hendra virus F. To confirm that the Hendra F residues K70 and K336 were responsible for decreased sensitivity to antibodies 4H3 and 1A9, we generated pseudoviruses expressing Hendra virus F with each of these sites separately mutated to their Nipah virus F identities (K70Q or K336R) and tested their neutralization relative to pseudoviruses expressing unmutated Nipah or Hendra F. Mutating these sites in Hendra F to the Nipah virus identities greatly sensitized pseudoviruses to neutralization, resulting in IC_50_s similar to unmutated Nipah F as expected from the deep mutational scanning (*SI Appendix*, Fig. S11*D*). These results suggest our deep mutational scanning in the Nipah F background can predict which antibodies neutralize Hendra virus and identify specific mutations responsible for differences in neutralizing potency.

## Discussion

Here, we measured the effects of all single amino acid mutations to Nipah virus F on two different protein phenotypes, cell entry and antibody neutralization. These data elucidate the constraints and evolutionary potential of this important protein, which is a key target of vaccines and therapeutics in development.

Our results show that Nipah F is less tolerant to mutations than RBP, mirroring the pattern observed in pairwise sequence identities, and more broadly with other viral entry proteins. For example, in deep mutational scanning experiments of influenza and SARS-CoV-2, which both use a single protein for binding and membrane fusion, the regions of the protein involved in fusion are generally more constrained than the regions that bind host receptors (40, 64). It appears that across diverse class I viral fusion proteins, membrane fusion is a more highly regulated and constrained phenotype than receptor binding.

Vaccine antigens based on stabilized viral entry proteins are a promising recent advance for increasing vaccine efficacy toward different viruses (60, 61, 65). Although some stabilizing mutations have been described for Nipah and Hendra virus F (29, 62, 66), we used our measurements of how mutations affect F-mediated cell entry to identify additional candidate stabilizing mutations. We focused on proline mutations in HRA due to prior work showing these mutations increase stability and do not disrupt protein folding (66). Although stabilizing mutations have been identified in other regions, such as HRB, we did not attempt to use our single-mutant deep mutational scanning data to identify candidate stabilizing mutations in HRB since stabilization in this region often requires multiple mutations (66). Other high-throughput approaches that specifically measure the effects of mutations on stability have been described for influenza virus hemagglutinin using pH as a selective agent (64, 67), since acidic pH triggers hemagglutinin-mediated fusion. Another alternative is cell-surface display approaches that directly screen for stability of the prefusion protein (68). But for our current Nipah virus F study, we were limited to using the cell entry data to identify candidate stabilizing mutations; so further experimental validation would be needed to confirm that these mutations actually increase prefusion F stability.

We also measured how all functionally tolerated F mutations affect neutralization by a panel of monoclonal antibodies, and found differences in how the neutralization by different antibodies was affected by F mutations. For instance, antibody 1F2 retained appreciable neutralization against a pseudovirus harboring F carrying the mutation that most impacted this antibody, but some other antibodies were completely escaped by single F mutations. Interestingly, some antibodies targeting HIV (69, 70) and influenza (71) have also been described that are less impacted by individual mutations. Antibodies that are more resilient to viral mutations are therefore promising candidates as Nipah virus prophylactics and therapeutics. Indeed, recent work has shown that prospective consideration of the effects of mutations during antibody development could have helped inform the development of more evolution-resistant antibody countermeasures during the COVID-19 pandemic (37).

Our study has several limitations. The experiments were performed with lentiviral pseudoviruses in cell culture. While the use of pseudoviruses obviates the substantial biosafety constraints of working with replicative Nipah virus, pseudoviruses in cell culture likely do not capture all the selective forces acting on mutations in vivo. Previous research on parainfluenza clinical isolates grown in cell culture demonstrated that viruses rapidly acquired substitutions in both RBP and F that altered fusogenicity (72). Our truncations to the cytoplasmic tails were necessary for effective pseudotyping, however the cytoplasmic tail of paramyxovirus RBP and F proteins have been implicated in cleavage, activation, expression, and triggering (73–76). Finally, our experiments focused on measuring the effects of single amino acid mutations to F, but in some cases the impacts of mutations during natural evolution can be modulated by epistatic interactions with other mutations (77, 78).

Overall, our study shows the Nipah F protein is more highly constrained than the RBP, making it an attractive target for vaccines and therapeutic antibodies. Our identification of highly constrained, surface-exposed residues can also help guide future attempts at defining RBP/F interacting regions, and quantifies the extent of functional constraint of different antibody epitopes. Taken together, these data shed more light on the biochemical function of F while also providing information that can help inform the development of future countermeasures to Nipah and other pathogenic henipaviruses.

## Materials and Methods

Deep mutational scanning experiments were performed with nonreplicative lentiviral-based pseudoviruses. Experiments were performed with CHO cells that stably express bat orthologs of the Nipah receptor (ephrin-B2 or -B3) from a natural henipavirus host, *Pteropus alecto.* All data, interactive visualizations, and raw data from experiments are publicly available on GitHub. The homepage (https://dms-vep.org/Nipah_Malaysia_F_DMS/) contains interactive visualizations to explore the deep mutational scanning data and links to additional datasets. The GitHub repository (https://github.com/dms-vep/Nipah_Malaysia_F_DMS) contains all code used to analyze the data, produce the figures, and information about available processed datasets. Additional *Materials and Methods* are available in *SI Appendix*.

## Supplementary Material

Appendix 01 (PDF)

## Data Availability

Data and scripts used for analyses have been deposited in GitHub (79). Previously published data were used for this work (Nipah RBP DMS GitHub repo (80)).
