# Supplementary material for: Functional and antigenic constraints on the Nipah virus fusion protein
Source: Proc Natl Acad Sci U S A. 2026 Feb 6;123(6):e2529505123. doi: 10.1073/pnas.2529505123 (PMC12885220; doi:10.1073/pnas.2529505123)
Supplement: Supplementary file 1 — Appendix 01 (PDF) [file pnas.2529505123.sapp.pdf]

PNAS

Supporting Information for

Functional and antigenic constraints on the Nipah virus fusion protein

Brendan B. Larsen[1], Sheri Harari[1], Risako Gen[2], Cameron Stewart[2], David Veesler[2,3], Jesse D. Bloom[1,3*]

[1]Basic Sciences Division and Computational Biology Program, Fred Hutchinson Cancer Center, Seattle, WA 98109, USA
[2]Department of Biochemistry, University of Washington, Seattle, WA 98195, USA
[3]Howard Hughes Medical Institute, Seattle, WA 98195, USA

*Corresponding Author: Jesse D. Bloom
Email: jbloom@fredhutch.org

This PDF file includes:

Supporting text

Figures S1 to S11

SI References

Supporting text
Methods and Materials

Data availability and interactive plots of results

All data, interactive visualizations, and raw data from experiments are publicly available on GitHub. The homepage (https://dms-vep.org/Nipah_Malaysia_F_DMS/) contains interactive visualizations to explore the deep mutational scanning data and links to additional datasets. The GitHub repository (https://github.com/dms-vep/Nipah_Malaysia_F_DMS) contains all code used to analyze the data, produce the figures, and information about available processed datasets. Sequencing data, including PacBio CCS and Illumina reads, have been deposited to the NIH SRA database under BioProject PRJNA1377637.

Biosafety

Due to the high biosafety level and restrictions on introducing novel mutations to authentic Nipah virus, we performed all of our experiments with lentiviral-based pseudoviruses in a biosafety-level-2 laboratory by trained individuals. Importantly, these pseudoviruses are non-replicative and do not encode any other viral proteins besides Nipah F, meaning that they are not fully replicative viral pathogens. The additional proteins required for virion formation are provided *in trans* on three separate plasmids (gag/pol, tat, rev) which limits the possibility of recombination generating a replication competent virus.

We also took steps to limit information on human-specific adaptations that could be potentially misused (1, 2). Rather than perform our experiments in the context of human cells or receptors, we used specific target cells as previously described (3). Briefly, we used CHO cells that stably express bat orthologs of the Nipah receptor (ephrin-B2 or -B3) from a natural henipavirus host, *Pteropus alecto*. This approach allows us to make measurements of the antigenic and functional effects of mutations that are beneficial for countermeasure development without providing information about mutations that might potentially adapt the F protein for entry in cells expressing human receptors.

Plasmids and primers

All plasmid and primer sequences used in this study can all be found here (https://github.com/dms-vep/Nipah_Malaysia_F_DMS/tree/main/data/paper_reference_files/sequences). All primers were ordered from Integrated DNA Technologies.

Monoclonal antibodies

Anti-F monoclonal antibodies 4H3, 2D3, 1A9, 2B12, 1F2 were synthesized by GenScript based on the original heavy and light chain sequences (4), which are also available here (https://github.com/dms-vep/Nipah_Malaysia_F_DMS/blob/main/data/paper_reference_files/sequences/antibodies/nipahF-antibody-sequences.csv). 12B2 was produced as previously described (5).

Cells

To produce lentiviral pseudoviruses from transfections, we used HEK-293T cells (ATCC Cat. No. CRL-3216). To produce pseudovirus from cell-stored libraries, we used HEK-293T-rtTA cells that express reverse tetracycline transactivator (rtTA) (6). HEK-293T cells were cultured in DMEM (Fisher, Cat. No. MT10013CV) supplemented with 10% Tet-free FBS (Fisher, Cat. No. A4736401), L-glutamine (Fisher Cat. No. MT25005CI), and Penicillin-Streptomycin (Fisher, Cat. No. MT30002CI).

For target cells for pseudovirus infections, we used CHO (ATCC, Cat. No. CRL-2242) cells or CHO clones expressing the bat (*Pteropus alecto*) ephrin-B2 (CHO-bEFNB2) and ephrin-B3 (CHO-bEFNB3) as previously described (3). CHO cells and bat ephrin stable CHO clones were cultured in Ham's F-12K (Kaighn's) Medium (Fisher, Cat. No. 21-127-030) supplemented with 10% FBS, Glutamine, and PenStrep. All cells were grown in incubators at 37°C with 5% CO2.

Cytoplasmic tail truncations in Nipah RBP and F
To obtain accurate measurements for deep mutational scanning, high pseudovirus titers are required to limit experimental bottlenecking of library diversity. Furthermore, due to the high fusogenicity of full-length Nipah RBP and F, high levels of syncytia form between 293T producing cells during viral rescue, which can scramble the genotype-phenotype linkage in individual virions. Therefore, we generated RBP and F cytoplasmic tail truncations (**Fig. S2A**) to boost titers (**Fig. S2B**) and reduce the amount of syncytia between pseudovirus producing cells. To determine the effect of F cytoplasmic tail truncations on cleavage and activation, we performed reducing SDS-page and western blotting (See section below *Western Blotting)* between full-length and the cytoplasmic tail truncation version of F. As expected, due to the removal of the endocytosis motif in the cytoplasmic tail (7), we observed decreased amount of cleaved F (F1) in the cytoplasmic tail truncation, however there were still appreciable amounts when we rescued viruses at 48 hours after transfection (**Fig. S2C**).

Creation of site-saturated single-mutant Nipah F libraries
We used the unmutated parental Nipah F sequence based on the reference sequence originally isolated in Malaysia during the first known outbreak in 1999 (GenBank accession NC_002728.1). We codon-optimized this sequence and removed 22 amino acids from the cytoplasmic tail at the C-terminus to improve lentiviral titers. The codon-optimized DNA sequence can be found here (https://github.com/dms-vep/Nipah_Malaysia_F_DMS/blob/main/data/paper_reference_files/sequences/unmutated_reference/NipahF_codon_optimized_CTdel_DNA.fasta).

To make the mutagenesis library we employed oligo pool (oPool) ssDNA synthesis offered by Twist BioSciences, which has an upper size limit of 300 nucleotides. The mutagenized region (sites 29-481, excluding the signal peptide and transmembrane domain) was split into six overlapping windows (**Fig. S3A**). Individual mutations at each site were designed with the most frequent human codon (For aspartic acid, we used GAT instead of GAC to limit introduction of BsmBI sites into mutant sequences) for all possible missense mutations using a custom jupyter notebook (https://github.com/dms-vep/Nipah_Malaysia_F_DMS/blob/main/notebooks/calculate_oPools.ipynb). Stop codons were designed to occur at every other position from sites 29 to 104. Each tile contained ~1,500 specific amino-acid mutations. In order to amplify each tile and perform Golden Gate Cloning from the oPool, we also included BsmBI sites in the correct orientation on both ends of the mutagenized fragment, and unique 20bp priming sites at the extreme 3' and 5' ends (**Fig. S3B**). Alignments of each designed window can be found here (https://github.com/dms-vep/Nipah_Malaysia_F_DMS/tree/main/data/paper_reference_files/sequences/designed_oPools).

PCR amplification of each tile separately from synthesized oPools
To amplify each tile separately from the delivered oPool, we used unique forward and reverse primers that matched the nucleotides added to the 3' and 5' ends of each tile (**Fig. S3B**). PCR primer sequences can be found here (https://github.com/dms-vep/Nipah_Malaysia_F_DMS/blob/main/data/paper_reference_files/sequences/primers/oPool_primers.csv). The PCR conditions are as follows.
25µL of 2x KOD Hot Start Master Mix (ThermoFisher, Cat. No. 71842-4), 2 µL of 10 µM of each primer, 1µL of oPool diluted DNA (0.25 ng/µL), and 20 µL of water. The thermocycler conditions were:

1. 95°C for 2 minutes
2. 95°C for 20 seconds
3. 70°C for 1 second
4. 62°C for 10s (ramp rate -0.5°C/second)
5. 68° for 25s
6. Return to step 2 for 19x cycles
7. 12°C hold.

PCR products were visualized on a 1% agarose gel to ensure each amplicon was successfully amplified and did not contain spurious bands. PCR products were cleaned with 1x AmpureXP beads (Beckman Coulter, Cat. No. A63881) and resuspended in Qiagen Buffer EB.

Golden Gate Destination Vectors

Following PCR amplification of the individual tiles, we cloned the amplicons directly in-frame into our lentiviral vector containing unmutated Nipah F. To prepare these vectors for Golden Gate Assembly, we domesticated our lentiviral plasmid (3137_pH2rU3_ForInd_mCherry_CMV_ZsGT2APurR) by changing two pre-existing BsmBI sites by primer mutagenesis. Next, we added BsmBI sites into the Nipah F open reading frame by primer mutagenesis separately for each window, so that each mutagenized tile could be cloned directly into the vector in-frame (**Fig. S3C**). The plasmid maps of the six destination vectors can be found here (https://github.com/dms-vep/Nipah_Malaysia_F_DMS/tree/main/data/paper_reference_files/sequences/destination_vectors).

Golden Gate Assembly of the amplified tiles and destination vectors

Each unique destination vector and amplified oPool were then combined in a Golden Gate Assembly reaction (**Fig. S3D**). The conditions are as follows. 1 µL destination vector (50fmol/µL), 1 µL PCR amplified oPool insert (100fmol/µL), 2 µL 10x T4 DNA Ligase Buffer (NEB, Cat. No. E1602S), 1 µL NEB Golden Gate Enzyme Mix BsmBI-v2 (NEB, Cat. No. E1602S), and 15 µL of water. Reactions were incubated in a thermocycler at 42°C for 1 hour, followed by 60°C for 5 minutes. Products were cleaned with 0.8x Ampure XP beads. 2 µL from each golden gate assembly reaction was electroporated into 10–beta electrocompetent cells (NEB, Cat. No. C3020K) with a BioRad MicroPulser Electroporator (Cat. No. 1652100), shocking at 2 kV, followed by resuspension of bacterial cells in 1mL of NEB 10-beta stable outgrowth media. An aliquot was diluted and plated onto LB ampicillin plates, with the remainder allowed to grow overnight at 37°C in 5mL LB and ampicillin. The total number of colony forming units (CFUs) for each unique pool ranged from 2.4e6 to 8e6, representing >1,000 CFUs per variant. High colony counts are necessary to prevent barcode swapping in subsequent steps caused by lentiviral recombination. The remaining library plasmids were extracted and purified from the overnight LB cultures with a QIAprep Spin Miniprep Kit (Qiagen, Cat. No. 27106). To ensure each mutagenized tile was inserted in-frame in the correct region, we sequenced full-length plasmids from two colonies from each reaction with Primordium.

Barcoding the mutant libraries

After confirming the plasmids were correctly assembled, we next added a random 16 nt barcode to each plasmid, which enables us to match each Nipah F mutation with a specific barcode as previously described (**Fig. S3E**) (3, 6).

The purified plasmid libraries were pooled equimolarly based on the DNA concentrations measured by a Qubit 4 Fluorometer (ThermoFisher, Cat. No. Q33238). Plasmids were digested at 37°C for 1 hour with XbaI (NEB, Cat. No. R0145S), gel extracted with a Nucleospin Gel and PCR Clean-up kit (Macherey-Nagel, Cat. No. 740609.5), followed by purification with 0.8x Ampure XP beads. To insert unique barcodes, we setup a HiFi reaction with the XbaI digested plasmid and a ssDNA barcoding oligo (5'-gcggaactccactaggaacatttctctctcgaatctagaNNNNNNNNNNNNNNNNAGATCGGAAGAGCGTCGTGTAGGGAAAGAG-3'). The HiFi conditions are as follows. 5 µL Barcoding oligo (0.2 pmol), 1 µL digested vector (10 fmol), 4 µL of water, and 10 µL of 2x HiFi MasterMix (NEB, Cat. No. E2621L). Reactions were incubated at 50°C for 1 hour, followed by electroporation into NEB 10-beta electrocompetent cells. To create separate replicate libraries, we used two separate HiFi reactions which became LibA and LibB. Electroporations were grown in LB and ampicillin overnight, with a small aliquot diluted to count the number of transformants. We obtained a total of 3.6e6 CFU for LibA, and 6.2e6 for LibB, which is >400 CFU per barcoded variant. As mentioned previously, the number of colonies needs to be significantly higher than the total size of the library to ensure enough unique barcodes are present to avoid swapping during lentiviral

recombination. After overnight growth, plasmids were extracted and purified with a Qiagen Spin MiniPrep kit.

Producing genotype-phenotype linked mutant pseudoviruses

We produced genotype-phenotype linked pseudoviruses as previously described(3, 6). Our method relies on coupling the genotype contained within the lentiviral genome with the mutagenized proteins on the surface of the pseudovirus. To accomplish this, we rescued VSV-G pseudotyped viruses containing the mutagenized lentiviral genome, followed by infection at low MOI to ensure each cell has no more than a single integrated provirus. This ensures that pseudoviruses produced from each individual cell are all genotype-phenotyped linked. Details of our method are as follows: we transfected HEK293T with the Nipah F library cloned into a lentiviral backbone (described above), plus three lentiviral helper plasmids (26_HDM_Hgpm227, 27_HDM_tat1b, 28_pRC_CMV_Rev1b), an expression plasmid for VSV-G (29_HDM_VSV_G), and BioT transfection reagent (Biolands Scientific, Cat. No. B01-02). Plasmid sequences can be found here: (https://github.com/dms-vep/Nipah_Malaysia_F_DMS/tree/main/data/paper_reference_files/sequences/plasmids). Supernatants were filtered through a 0.45 µm syringe filter (Corning, Cat. No. 431220) after 48 hours and titered on HEK293T-rtTA(6) cells to estimate an appropriate MOI. HEK293T-rtTA cells were then infected with pseudoviruses expressing VSV-G at an MOI of <0.01 to ensure each cell contained no more than a single integrated lentivirus. Cells were then passaged in the presence of 1 µg/mL puromycin (ThermoFisher Cat. No. A1113803) until all cells were ZsGreen positive. These cell-stored libraries were frozen down with 10% DMSO and stored in the gas phase of liquid nitrogen.

To generate the library virus used for infections, we thawed a fresh aliquot of cells and cultured them until they could be plated onto multiple five-layer flasks (Corning Falcon 875cm2 Rectangular Neck Cell Culture Multi-Flask, Cat. No. 353144). The following day, we transfected the cells. To generate VSV-G expressing pseudoviruses from the library, we prepared the following transfection mix. 45 µg each of the three helper plasmids (gag/pol, tat, rev), 15 µg of VSV-G expression plasmid, 7.5 mL of DMEM, and 225 µL of BioT transfection reagent. Transfection mixtures were incubated for 10 minutes at room temperature and added to each five-layer flask. 48 hours later, supernatants were filtered through a 0.45 µm SFCA Nalgene 500 mL Rapid-Flow filter unit (Cat. No. 09-740-44B).

To produce the RBP_{wt}/F_{mutant} pseudovirus libraries, the same approach above was used, except we used 48.75 µg of each helper plasmid, and 3.75 µg of unmutated Nipah RBP (3336_HDM_Nipah_RBP_CTdel, RBP_{wt}) that was codon-optimized and contained a 32 amino-acid truncation in the N-terminal cytoplasmic tail(3). Titers of unconcentrated library pseudovirus were typically around $5*10^4$ transducing units (TU)/mL. Following filtration of the RBP_{wt}/F_{mutant} pseudovirus libraries, we concentrated them to obtain high titers needed for our selections.

We used two different methods to concentrate the lentiviruses for selections, which did not differ in their concentration efficiency or affect the results. We mixed virus supernatant and Lenti-X Concentrator (Takara, Cat. No. 631232) at a 3:1 ratio and incubated overnight at 4°C. Tubes were then spun at 1500g for 45 minutes at 4°C. Supernatants were discarded, and pellets were resuspended in complete F-12K media. Alternatively, we added 50mL of virus supernatants to Pierce Protein Concentrators, 100k MWCO (Thermo, Cat. No. 88537) and spun at 2000g for 30 minutes. Liquid at the top of the filters containing the concentrated pseudoviruses was transferred to new tubes. All concentrated viruses were stored in aliquots at -80°C. Following concentration, titers were typically around $1*10^6$ TU/mL.

Library infections and DNA template recovery

To obtain DNA templates from lentiviruses for PCR amplification and sequencing, we used the following approach as previously described (3, 6). Briefly, our method relies on obtaining unintegrated lentiviral DNA that is generated by lentiviral reverse transcription. This serves two main purposes. First, most HIV DNA templates do not integrate into the host genome following infection. Second, if we attempted to extract provirus from cell gDNA, it would decrease PCR efficiency. Therefore, we infect target cells with $1\text{-}2x10^6$ TU of library virus and 8-12 hours later extract low molecular weight DNA using a Qiagen QIAamp Spin Miniprep kit. For VSV-G

pseudotyped lentiviruses, we infected CHO cells plated in six-well dishes, for RBP_{wt}/F_{mutant} pseudoviruses we either used CHO-bEFNB2 or -bEFNB3 cells. 8-12 hours later, media was removed from the cells, and 450µL of trypsin was added. After 5 minutes, 550µL of complete F-12K media was added and mixed to dislodge all the cells. Cells were pelleted at 300g for 4 minutes. Supernatant was removed and cells were resuspended in 250µL of P1 Buffer. Cell suspensions were then miniprepped to isolate non-integrated lentiviral templates.

Long-read sequencing to link mutations to barcodes
To link the 16 nt random barcodes with each mutation, we performed long-read PacBio sequencing of lentiviral DNA templates that were miniprepped from target cells 8-12 hours after infection with the concentrated VSV-G expressing library virus as previously described (3, 6). Due to the pseudodiploid nature of lentiviruses and possible recombination, the linkage of barcodes and mutations is performed after generating the cell libraries to ensure the linkage is accurate. Our approach here includes low PCR cycles to minimize strand exchange, while also quantifying the amount of strand exchange during PCR, which artificially scrambles the barcode-mutation linkages. Specifically, we included single nucleotide tags (either 'G' or 'C') within the first round primers. The amplicons from the first round PCRs are then pooled and undergo a second round of PCR. Strand exchange that occurred during the 2nd round of PCR is identified by comparing the single nucleotide tags at the 5' and 3' ends, which were introduced in the first round PCR.

1st round PCRs were done in three separate reactions to add either a G or C at the 5' and 3' ends to allow identification of strand exchange. 1st round PCR conditions were as follows. 20 µL of KOD Hot Start Master Mix, 10µL of DNA templates from minipreps, 1µL each of 10µM primers (PacBio_5pri_G + PacBio_3pri_C) or (PacBio_5pri_C + PacBio_3pri_G), and 8µL of water. The thermocycler conditions were:

1. 95°C for 2 minutes
2. 95°C for 20 seconds
3. 70°C for 1 second
4. 60°C for 10 seconds with a ramp rate of -0.5°C/sec
5. 70°C for 1 minute
6. Return to step 2 for 7x cycles
7. 12°C hold

PCR products from three separate first round PCR were pooled and cleaned up with 1x Ampure XP beads. The conditions for the 2nd round PCR are as follows. 25 µL of KOD Hot Start Master Mix, 2 µL of each primer at 10 µM (5'_PB_Rnd2 and 3'_PB_Rnd2), and 21 µL of cleaned round 1 product. The thermocycler conditions were:

1. 95°C for 2 min
2. 95°C for 20 seconds
3. 70°C for 1 second
4. 60°C for 10 seconds with a ramp rate of -0.5°C/sec,
5. 70°C for 1 minute.
6. Return to step 2 for 10x cycles
7. 12°C hold

PCR products were then cleaned with 1x Ampure XP beads, followed by circular consensus sequencing on a PacBio Sequel IIe. We found that 2-3% of all reads had strand exchange, as identified by comparing the single nucleotide tags described above.

Illumina barcode sequencing
To recover barcode frequencies associated with each variant, we performed Illumina sequencing on amplified unintegrated viral template DNA extracted from target cells 8-12 hours after infection (see section *Library infections and DNA template recovery*). Next, we set up PCRs for each selection experiment with the following conditions. 25 µL of KOD Hot Start Master Mix, 1.5 µL each of forward and reverse 1st round Illumina primers (Illumina_Rnd1_For and

Illumina_Rnd1_Rev3), and 22 µL of the miniprepped DNA templates. The thermocycler conditions were:

1. 95°C for 2 minutes
2. 95°C for 20 seconds
3. 70°C for 1 second
4. 58°C for 10 seconds with a ramp rate of -0.5°C/sec
5. 70°C for 20 seconds
6. Return to step 2 for 27x cycles
7. 12°C hold

Following 1st round PCRs, we measured DNA concentrations using a Qubit 4 Fluorometer. DNA concentrations were normalized and then added to 2nd round PCRs each containing 2 µL of 10 µM unique indexed 2nd-round primers, which is used to identify index-hopping that can occur during Illumina sequencing(8). PCR conditions were similar to above, except only 1 µL of 1st round PCR was used as template, with the remaining volume containing water, and only 20 PCR cycles were used for the 2nd round PCRs. Next, the indexed 2nd round samples were pooled equimolarally and run out on a 1% agarose gel. DNA from bands of the correct size were excised from the gels and extracted with a Nucleospin Gel and PCR Clean-up kit. The DNA was then cleaned with a final purification step using 0.8x Ampure XP beads and washed three times with 80% EtOH. Pooled samples were then sequenced on an Illumina NextSeq with a P2 kit, or a NovaSeq lane for 50 cycles, depending on how many multiplexed, indexed samples we included. We typically received 20-100 million reads per sample, corresponding to >500x coverage of each variant.

Analysis of PacBio sequencing and creation of barcode-variant lookup table

For processing the sequencing data from the deep mutational scanning experiments, we used the *dms-vep-pipeline-3 v3.25.0* package (https://github.com/dms-vep/dms-vep-pipeline-3), which is briefly described here. To link specific mutations with each barcode, we performed PacBio circular consensus sequencing (CCS) on amplicons spanning the entire F protein and the 16 nt barcodes (see section *PacBio sequencing*).

From the PacBio CCS data obtained for both libraries, we first aligned the reads to the unmutated Nipah F reference sequence using the *alignparse* package (9). Reads that aligned poorly or had a higher than expected number of mutations in the unmutated regions were filtered out. Next, variants that did not contain a barcode or were the result of strand exchange were filtered out. Consensus sequences for each barcode/variant sequence were constructed using *alignparse*, while requiring a minimum of at least three CCS reads and a max cutoff of 0.2 for any minor variants within the consensus. The final barcode/variant lookup tables were used as a reference for all downstream analyses that used the short-read Illumina sequencing of the barcodes only.

Calculation of functional scores

To calculate the efficiency of cell entry of different variants relative to the unmutated sequence, we compared variant frequencies derived from infecting cells with either VSV-G or RBP_{wt}/F_{mutant} pseudotyped viruses as previously described(6). Here, the VSV-G condition serves as a 'control', and allows us to determine frequencies of variants contained within the lentiviral genome that would be highly deleterious for F-mediated entry. From Illumina sequencing of barcodes recovered from these infections, we obtain relative frequencies of each infecting variant.

Illumina sequencing data were first filtered to ensure that all bases had a minimum sequencing quality score of 20, and were then aligned to the barcode variant table generated from the PacBio CCS described above. Barcode sequences below a frequency $2*10^{-5}$ were excluded from downstream analyses.

We next compared the frequency of barcodes between the VSV-G and RBP_{wt}/F_{mutant} selections using the package *dms_variants* (https://github.com/jbloomlab/dms_variants) as previously described (3, 6). Briefly, functional scores were calculated using enrichment ratios: $\log_2([n^v_{post} / n^{wt}_{post}] / [n^v_{pre}/n^{wt}_{pre}])$ where n^v_{post} and n^{wt}_{post} are the counts of variant *v* or the

unmutated variants from the Nipah F pseudovirus infection, respectively. The variant *v* or unmutated counts in the VSV-G pseudotyped infection are n^{v}_{pre} and n^{wt}_{pre}, respectively. The lower limit of detection for mutation effects was approximately -4, which corresponds to the median effects of stop codons, which should result in non-functional protein. Therefore, we clipped the functional scores below this threshold and used those functional scores for our cell entry calculations.

Calculation of mutation effects on cell entry

Although our libraries primarily contain variants with a single mutation relative to the unmutated parental strain (69%), a subset of our library contains multiple amino-acid mutations. To decompose the effects of these multi-mutants, we utilized the *multiDMS* package to apply global epistasis fitting (10, 11). We compared the effects of the decomposed effects with the uncorrected functional scores of single-mutations only (**Fig. S5**). These comparisons were all well-correlated, with outlier mutations generally having few (<2) barcodes associated with a single mutation. Thus, for all downstream analyses, we used the decomposed functional effects produced from the global epistasis modeling.

To generate the final cell entry effect values for each mutation, we performed a total of two technical replicates from two separate rescues for each library, for a total of eight functional selections (four each from LibA and LibB). The effects for each mutation were then averaged from the eight functional selections. To filter out low quality or noisy data, we applied two filters. First, we required each mutation to occur with two unique barcodes (*times_seen >= 2*). Second, we removed any mutation that had a high standard deviation between replicates (*effect_std <= 1*). The final functional effect values reported in the figures correspond to the average effect across libraries and replicates.

Effects of mutations on antibody neutralization

To measure the effects of mutations on antibody neutralization, we used a previously described method (6) with a few modifications described here. ~$1x10^6$ CHO-bEFNB3 cells were plated in individual wells of a 6-well plate. The following day, ~$1x10^6$ TU of library virus was either added directly onto the cells (no-antibody control) or were incubated in the presence of antibody for one hour prior to adding to cells. Antibody concentrations were selected that generally corresponded to a range at which 50% of variants were neutralized, up to 99.5%. During DNA template extraction, we spiked in DNA plasmid containing eight known barcodes that would correspond to ~1% of the reads in the no-antibody control. This DNA plasmid spike-in allowed us to estimate the amount of neutralization each antibody condition had relative to the no-antibody control, as previously described (6).

Following extraction and sequencing of the barcodes, we parsed, filtered, and aligned the barcodes as described in the previous section. We calculated an escape score as the $\log_2$ transformed values of: $(F * [n^{v}_{post} * N_{pre}] / [n^{v}_{pre} * N_{post}])$ where F is the overall fraction of the library that escapes neutralization, which is derived from the known DNA barcodes that we spiked in during template extraction. n^{v}_{post} and n^{v}_{pre} are the counts of variant *v* in the antibody condition and no-antibody condition, respectively. N_{pre} and N_{post} are the summed counts of all variants in the no-antibody control and antibody condition, respectively. We then fit neutralization curves for each selection as implemented in the package *polyclonal* (https://jbloomlab.github.io/polyclonal/) (12). We filtered the antibody escape data using three different cutoffs. First, we required mutations to occur with two unique barcodes (*times_seen >=2).* Second, since we can only measure mutations that have at least some level of cell entry, we excluded measurements of mutations with very low cell entry scores (*min_func_effect >= -2.5).* Third, we filtered out mutations that had high standard deviations between replicates (*escape_std_dev <= 2).* Reported escape scores for each mutation are the average effect calculated from at least two different independent selections with LibA and LibB.

Validation of mutation effects using individual pseudoviruses

To validate the effects of mutations on cell-entry and antibody neutralization, we generated a set of plasmids expressing Nipah F that contained different single mutations. The parental Nipah F sequence is identical to the unmutated Nipah F sequence used in the lentiviral vector described

above, but instead placed into a mammalian expression vector (5073_HDM_NipahF_CTdel_GeneArt). All single amino-acid mutations were generated by primer mutagenesis followed by sequencing confirmation by Primordium.

For cell entry validations, we selected mutations spanning a range of entry effects, which include K98R, L104N, V108D, R109Q, G215R, and E361G, which corresponded to a range of predicted cell entry effects. We made three separate plasmid preps of each Nipah F validation mutation and transfected them into 293T cells, along with plasmid 2727_pHAGE6_Luciferase, 26_HDM_Hgpm2, and 3336_HDM_Nipah_RBP_CTdel. 48 hours later, supernatants were filtered to remove cell debris. CHO-bEFNB3 cells plated on a 96-well plate were infected with dilutions of virus supernatant for each mutation and replicate, and Luciferase measurements were taken 48 hours later on a plate reader using a Bright-Glo Luciferase Assay System (Promega, Cat. No. E2620). We then checked to make sure our readings were within a linear range and compared the average luciferase signal of each mutation to the unmutated Nipah F readings.

To validate the magnitude of escape among antibodies, we generated the top escape mutation that had a cell entry score greater than -1 for each antibody in the same mammalian expression vector described above. These mutations were V159R (12B2), A165L (2D3), V65D (4H3), T43P (1A9), T286W (1F2), and E406T (2B12). To validate 4H3 escape measurements, we made mutations corresponding to Nipah F T54E, K98R, T250I, T250W, D255E, F282T, and N350E. We used a luciferase-based system identical to the cell entry assay described above with the following differences. Filtered virus supernatants were incubated with different concentrations of antibody for one hour prior to adding to 96-well plates containing CHO-bEFNB3 cells. Luciferase measurements were taken identical to above. Relative luciferase readings compared to wells without antibody were used to generate neutralization curves. Neutralization assays were performed in duplicate, unless noted otherwise.

Western Blotting

To determine the effects of the cytoplasmic tails truncations on F cleavage and RBP/F incorporation into pseudoviruses, we performed reducing SDS-PAGE followed by western blotting. To generate the pseudovirus, we transfected 15cm plates containing 293T cells with 15 µg of plasmid 2727_pHAGE6_Luciferase, 10µg of plasmid 26_HDM_Hgpm2, 45 µL BioT, and combinations of RBP+F plasmids (2.5µg). 30 hours later, supernatants were filtered through a 0.45 µm syringe filter (Corning, Cat. No. 431220), followed by ulltracentrifugation at 100,000 x g for 1 hour. Pellets were resuspended in 500 µL of PBS. An aliquot of the concentrated virus was mixed with 5x Pierce Lane Marker Reducing Sample (ThermoFisher Cat. No. 39000) and boiled for 3 minutes. 10 µL of protein or 5 µL of marker (BioRad Precision Plus Protein Dual Color Standards; Cat. No. 1610374) were loaded onto SDS-PAGE gels (4-20% Mini-PROTEAN TGX Precast Protein Gels, 10-well, 50 µL; BioRad Cat. No. 4561094) and run at 100V for 1.5 hours. Gels were transferred with iBlot3 Transfer Stacks Mini PVDF (Invitrogen Cat. No. IB34002). Membranes were blocked for 2 hours with 5% milk in 1X Tris Buffered Saline with Tween 20. Primary antibodies were then used to stain for p24 at 1:4000 (Rabbit anti-HIV1 p24 antibody; Abcam Cat. No. ab32352), RBP at 1:3000 (Rabbit pAb Nipah virus Glycoprotein G; SinoBiological Cat. No. 40980-T62), or F at 1:3000 (Rabbit Anti-Nipah Fusion F0 polyclonal antibody; Antibody system Cat. No. PVV08101). After 2 hours, membranes were washed 3x with TBST, followed by incubation with a goat anti-rabbit HRP secondary antibody (Invitrogen Cat. No. 31460) at 1:5000. After 1 hour of gentle shaking, membranes were washed 3x with TBST and 2X with PBS, followed by developing with SuperSignal West Pico PLUS Chemiluminescent Substrate (ThermoScientific Cat. No. 34580). After 5 minutes, membranes were imaged and analyzed in ImageJ.

Sequence analysis of publicly available sequences

To generate sequence alignments, we downloaded all complete whole genomes from Nipah and Hendra viruses from GenBank (accessed May 6th, 2025). GenBank accessions of all sequences can be found here (https://github.com/dms-vep/Nipah_Malaysia_F_DMS/tree/main/data/paper_reference_files/sequences/genbank). Sequences were aligned using MAFFT v7.520 (13), and maximum-likelihood phylogenetic trees were inferred with IQ-Tree v2.2.2.6 (14). Alignments corresponding to RBP and F were extracted

using Geneious Prime v2024.0.7. Amino-acid polymorphisms were calculated from the alignments using a custom jupyter notebook (https://github.com/dms-vep/Nipah_Malaysia_F_DMS/blob/main/analysis/workflow/notebooks/find_variable_sites_from_alignments.ipynb). Phylogenetic trees were visualized with the *baltic v0.3* package (https://github.com/evogytis/baltic).

Structural analyses

All protein structures were visualized with ChimeraX v1.10 (15) using publically available structures deposited in the Protein Data Bank (PDB). For the Nipah F prefusion structure, we used PDB 5EVM. For the antibody structures, we used PDBs 7UPK (1A9), 7UPD (2B12), 7UP9 (2D3), 7UOP (4H3), and 7KI4 (12B2). For 1F2, there is not a structure available, but based on previous low-resolution cryo-EM, the binding interface is very similar to antibody 1H8 (7UPA) (4), which was used for 1F2 footprint estimates. The Nipah virus F postfusion structure was generated with AlphaFold2 (16) through CollabFold (17) using the postfusion structure of Langya virus F (8TVE) (18) as a template before removing residues with the lowest confidence from the model manually.

Identification of candidate prefusion stabilizing mutations

To identify candidate prefusion stabilizing mutations, we reasoned that sites with highly deleterious proline mutations in regions that undergo large conformational changes between the pre- and postfusion conformations restrict the transition to the postfusion conformation, which is necessary for cell entry. However, if all or most other mutations at a site are deleterious, the unmutated residue is likely critical for correct protein folding and is not a good candidate for introducing stabilizing mutations. We used the cell entry deep mutational scanning data to identify sites meeting specific filtering criteria, with the rationale for each given below. We only included sites within the apical domain of prefusion F, since it undergoes the largest change between pre- and postfusion conformations (19), and previously described stabilizing mutations were all located in the apical domain (20, 21). We excluded sites in the fusion peptide (sites 110-125) since they are likely important for membrane fusion. Additional criteria included the site could not be in a helix or sheet in the prefusion structure, or at a residue that is involved in hydrogen bonding with sidechains in nearby residues, as these sites are likely important for maintaining correct protein folding. We required the proline mutation to have a cell entry score <-3, and the site needed at least four mutations with a cell entry score >-1. Finally, the site could not be at a N-linked glycosylation site or cause a disruption in a glycosylation site (i.e. a proline mutation at X in the NX(T/S) glycosylation motif).

Supplemental Figures

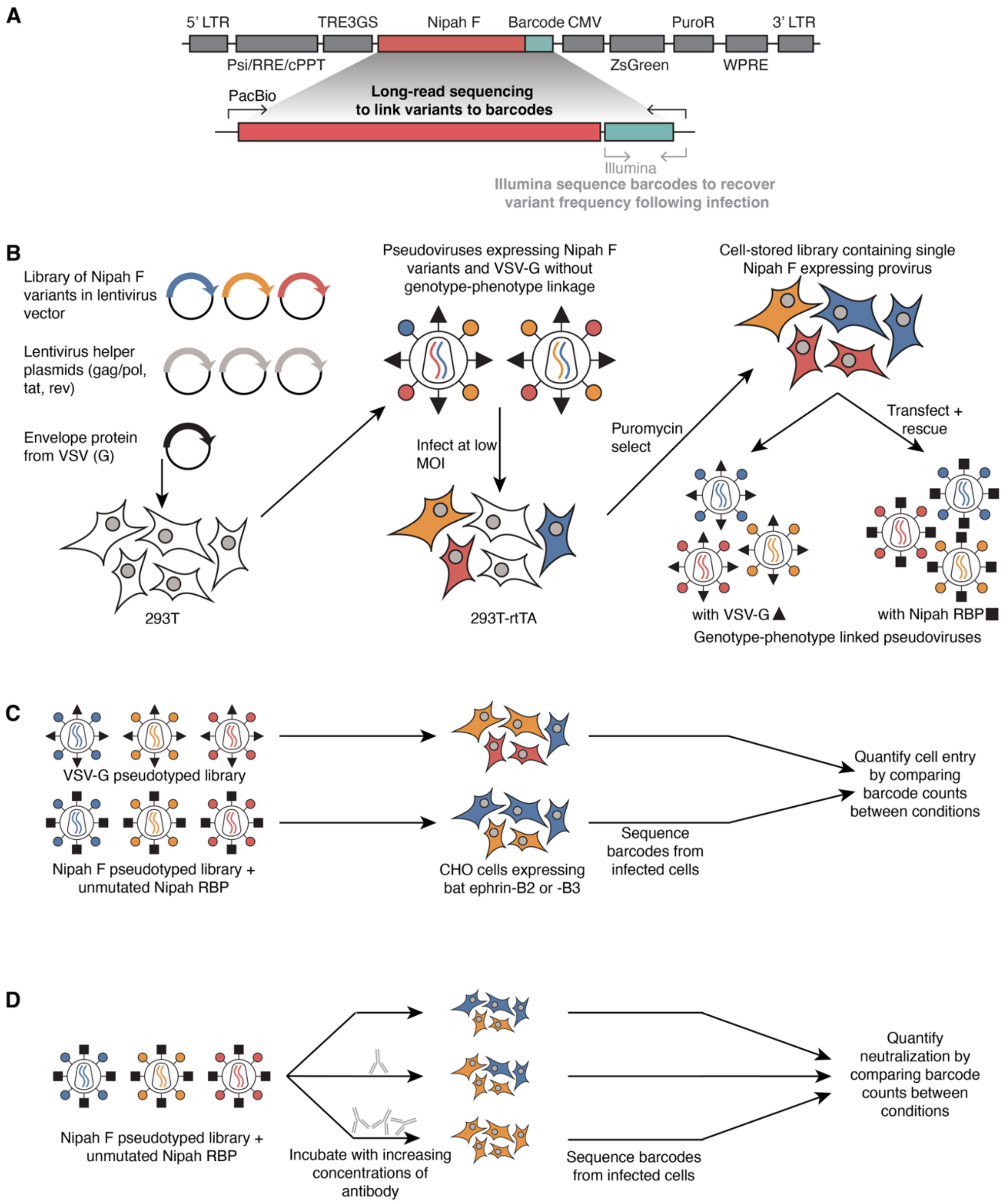

Fig. S1. Graphical overview of the lentiviral backbone, production of lentivirus libraries, and selection strategies for generating deep mutational scanning data.
A) Lentiviral backbone containing the Nipah F coding sequence (with a cytoplasmic tail truncation), essential lentiviral elements (long-terminal repeats, psi packaging element, rev response element, and central polypurine tract), and selection markers (ZsGreen and puromycin resistance). The F open reading frame is followed by a random unique 16-nucleotide barcode, which allows us to link specific F mutations with barcodes by long-read PacBio CCS sequencing. Downstream experiments rely on Illumina sequencing of the barcodes to obtain frequencies of

the variants within each library. **B)** Creation of genotype-phenotype linked pseudovirus libraries. Lentivirus plasmid libraries containing all possible F amino acid mutations in the ectodomain (sites 29-481) are transfected into 293T cells with other lentiviral helper plasmids (gag/pol, rev, tat) and the envelope protein from the vesicular stomatitis virus (VSV-G), which has broad tropism. Pseudoviruses rescued from transfections cannot be used for deep mutational scanning due to mismatches between the genotype encoded in the pseudodiploid virion and the protein variants expressed on the surface. Instead, these pseudoviruses are used to infect 293T cells at a low multiplicity of infection (MOI < 0.01) to ensure a single integrated variant per cell, and bottlenecked to only get ~50,000-70,000 unique variants. Cells are passaged in the presence of puromycin, creating a cell-stored library of Nipah F variants contained within a lentiviral backbone. Pseudovirus libraries are rescued from these cells by re-transfecting essential lentiviral helper plasmids (gag/pol, tat, rev) and either VSV-G or the Nipah virus receptor binding protein. The final pseudovirus libraries express Nipah F variants with either VSV-G or the unmutated Nipah receptor binding protein. These libraries are used in the following steps to measure the effects of mutations. **C)** Overview of selection experiments used to obtain the effects of mutations on cell entry. Libraries generated in (B) that express Nipah F variants with either VSV-G or the unmutated Nipah virus receptor binding protein are used to infect stable CHO cells expressing either bat ephrin-B2 or -B3. Pseudoviruses with VSV-G on their surface will infect cells regardless of the F variant displayed and acts as a control for measuring the baseline composition of variants in the library. Pseudoviruses with the Nipah receptor binding protein and the F variants will only enter cells if they are functional. Twelve hours after infection, unintegrated lentiviral DNA templates are extracted from cells and undergo PCR and Illumina sequencing. Barcode frequencies are used to estimate the relative composition of the variant libraries in the different conditions and estimate the effects of specific mutations on cell entry. **D)** Overview of selection experiments used to obtain the effects of mutations on antibody neutralization. Pseudoviruses expressing the unmutated Nipah receptor binding protein and F variants are used to infect CHO cells expressing bat ephrin-B3. Pseudovirus libraries are either incubated with varying amounts of antibody or added directly to cells (as the control). Variant frequencies are obtained by extracting unintegrated lentiviral template DNA from the cells, amplifying the barcodes with PCR, and sequencing with Illumina. To obtain estimates of the amount of libraries that are neutralized, plasmid DNA containing known barcodes are spiked-in during DNA extraction.

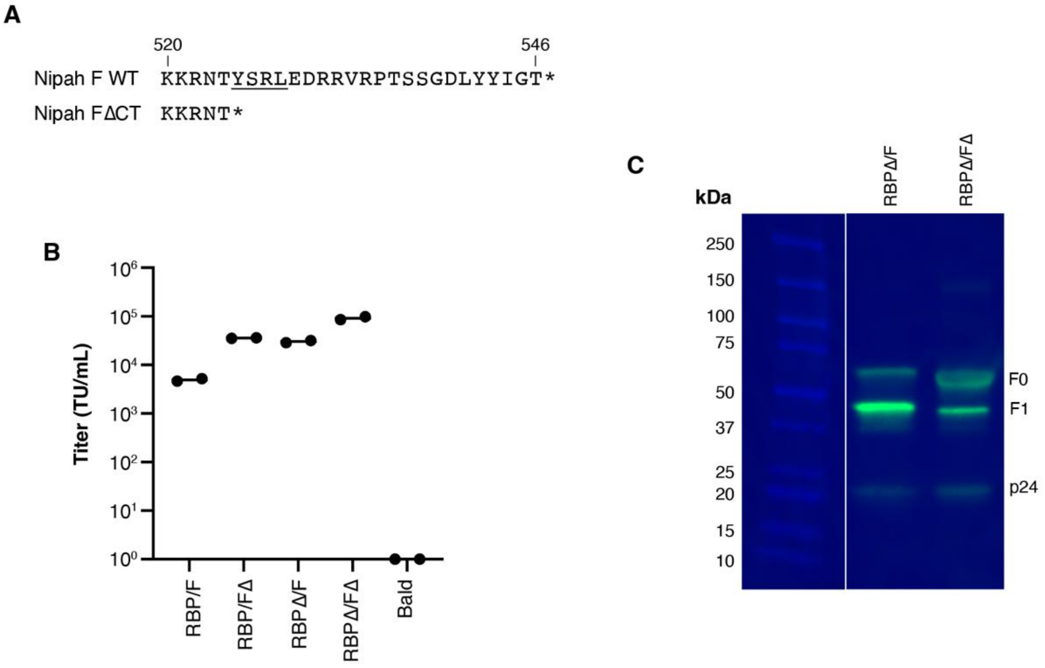

Fig. S2. F cytoplasmic tail truncation affects pseudovirus titers and cleavage
A) Amino-acid alignment of wildtype (WT) and 22 amino-acid cytoplasmic tail deletion (∆CT) at the C-terminus of Nipah virus F. Endocytosis motif ‘YSRL’ is underlined. **B)** Pseudovirus titers in CHO-bEFNB3cells of different combinations of RBP and F constructs with or without the cytoplasmic tail deletion. **C)** Reduced SDS-PAGE western blot on F and p24 in pseudoviruses (see Methods for details on staining). F0 and F1 are uncleaved and cleaved products, respectively. Gel is representative of two experiments.

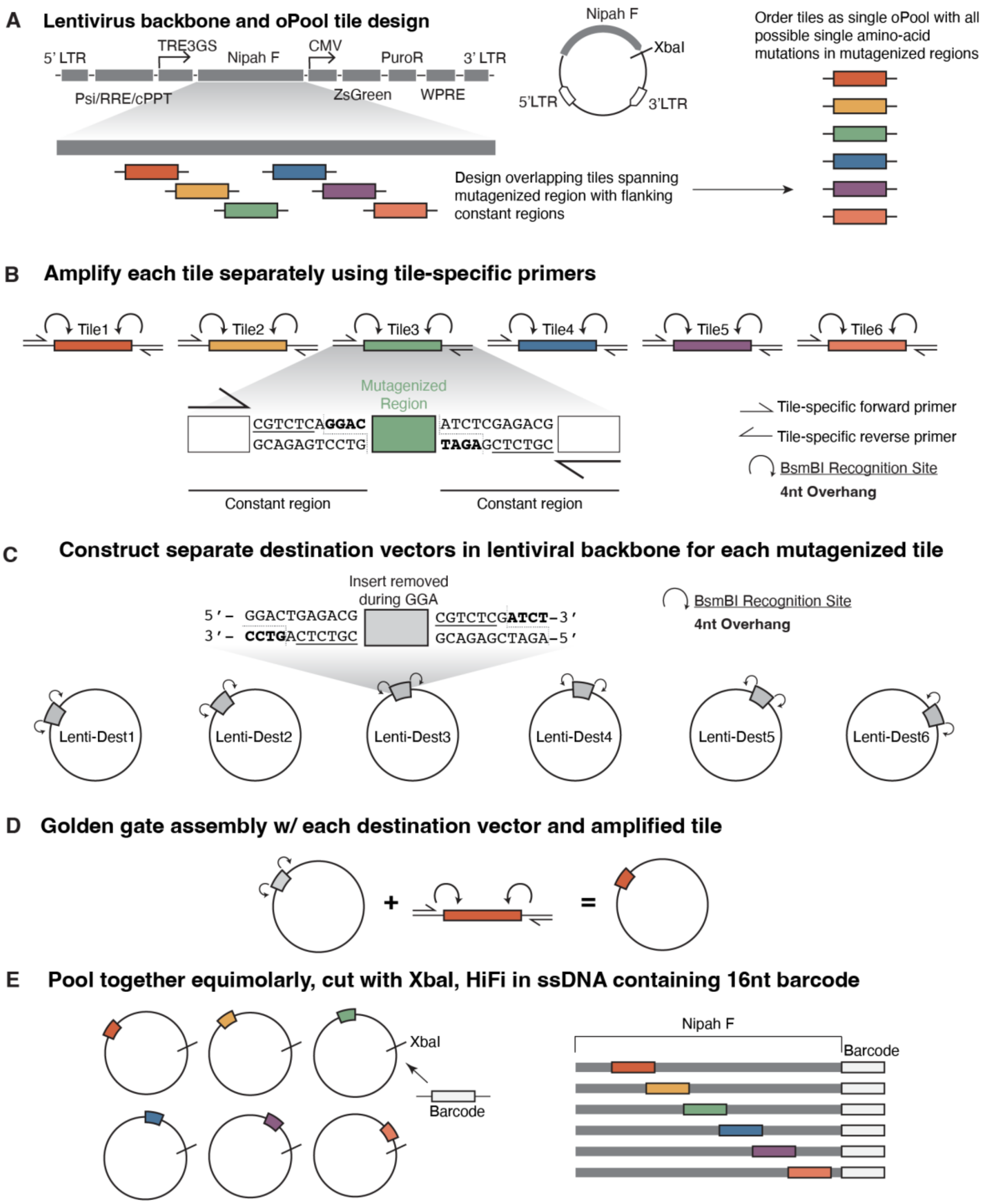

Fig. S3. Schematic of single-mutation library construction using Twist oPools and Golden Gate Assembly.

A) Plasmid map shows the different open reading frames of the parental F sequence contained downstream of a TRE3GS promoter within a lentiviral genome. The 3' LTR has been repaired so that lentiviral templates can be produced from provirus. Sites corresponding to amino acids 29-481 in Nipah virus F were divided into six overlapping tiles each spanning roughly ~220 nucleotides. Mutations to all possible amino-acid mutations at each position in the tile were constructed using the most frequent human codon. Finally, to facilitate downstream amplification of specific pools and cloning, constant regions were added to the 5' and 3' ends of each

mutagenized window and contain BsmBI sites and unique forward and reverse primer sites. Each tile contains ~1500 mutations and was synthesized by Twist BioSciences. **B)** Each oPool tile was amplified with specific, unique primers that matched the unique priming site sequences included in the oPool design. **C)** We constructed six separate destination vectors based on the parental lentivirus genome in (A). We added inward-facing BsmBI sites at the flanks of each window using primer mutagenesis that result in 4 nt overhangs that match the overhangs in the tiles. **D)** Each amplified tile was mixed with the specific destination vector and assembled with Golden Gate Assembly. E) Once the tiles were cloned into each destination vector, we barcoded each plasmid. First, the six plasmids were mixed equimolarlly, followed by cutting with XbaI which is just downstream of the Nipah F ORF. HiFi reactions were done with the cut plasmids and a single stranded DNA oligo with flanks matching each side of the XbaI cut site and a 16nt random sequence in the middle.

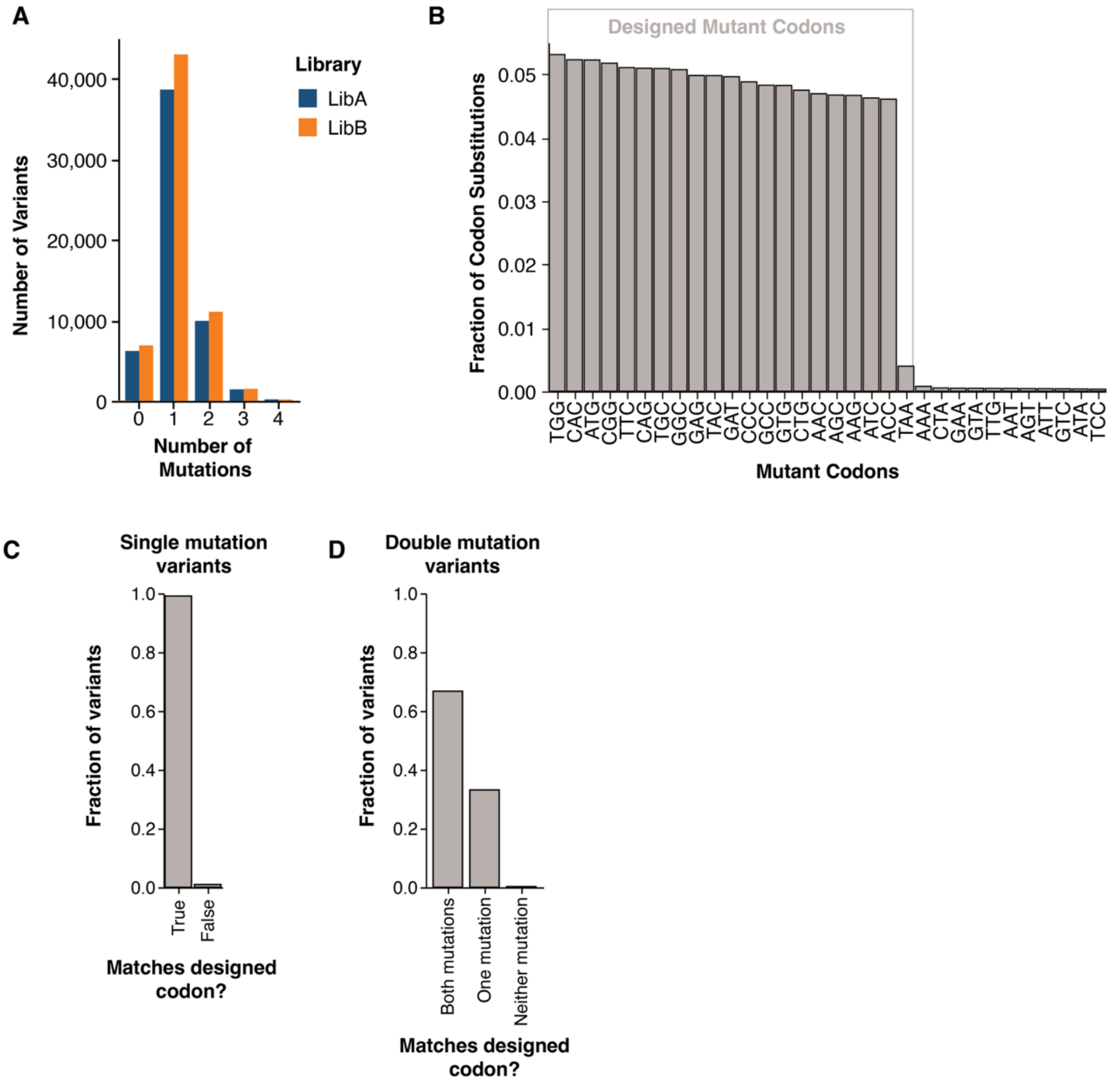

Fig. S4. Deep mutational scanning library statistics.
To link specific mutations with 16nt barcodes, we sequenced the full F open reading frame with PacBio along with the barcode downstream of the stop codon. Templates for sequencing were generated by rescuing pseudoviruses from cell-stored libraries expressing VSV-G and used to infect CHO cells. 12 hours later, unintegrated DNA viral templates were isolated and purified using a Qiagen Spin MiniPrep Kit, which were then used in PCR to generate amplicons for PacBio sequencing. **A)** Number of amino-acid mutations relative to the parental strain found in each variant. **B)** Frequency of mutated codons present in both libraries, with the specific codons used in oPool synthesis surrounded by a black box. There were 19 total missense codons at each site, and a subset of sites were mutated to a stop codon (TAA). **C)** Fraction of variants with one mutation relative to the parental strain that match the exact codon used for oPool design. **D)** Fraction of variants with two mutations that match the codons used for oPool design. The majority of variants with two mutations match the codons used for oPool design at both sites, suggesting they are generated by lentiviral recombination rather than errors in oligo synthesis.

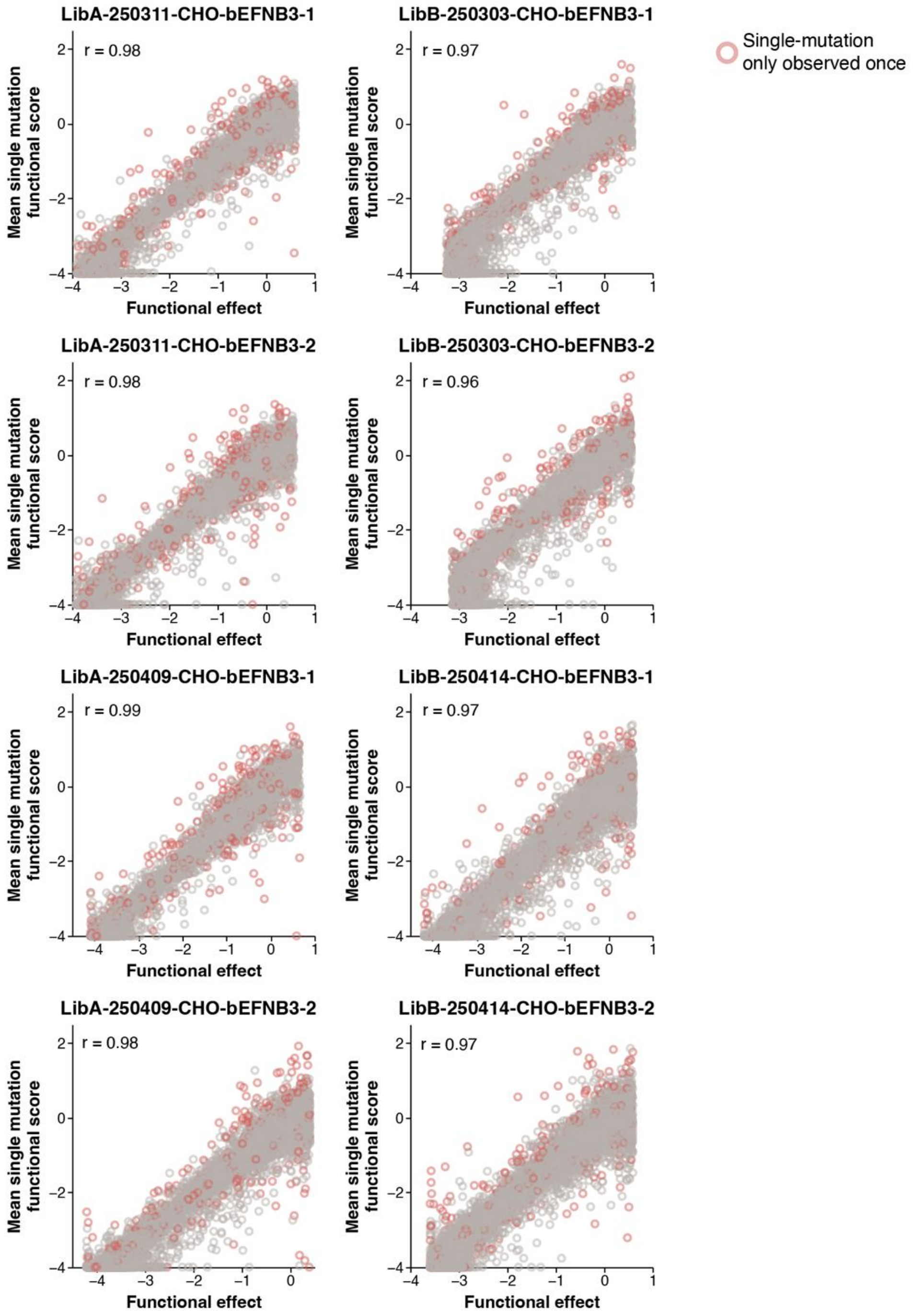

Fig. S5. Correlation between functional scores from single-mutation variants and the decomposed functional effects following global epistasis fitting for four replicate selections each from LibA and LibB.
Functional scores for each variant are calculated as the difference in frequency between the VSV-G and RBP/F pseudovirus infections. Functional effects are calculated by fitting a global epistasis model to decompose the effects of multiple mutations (1). Each plot shows an independent selection in CHO-bEFNB3 cells, and the r value is the Pearson correlation coefficient. Red points are mutations that are only linked to a single barcode and represent low-confidence measurements. Data were clipped at -4, corresponding to the lower range of detection for our assay.

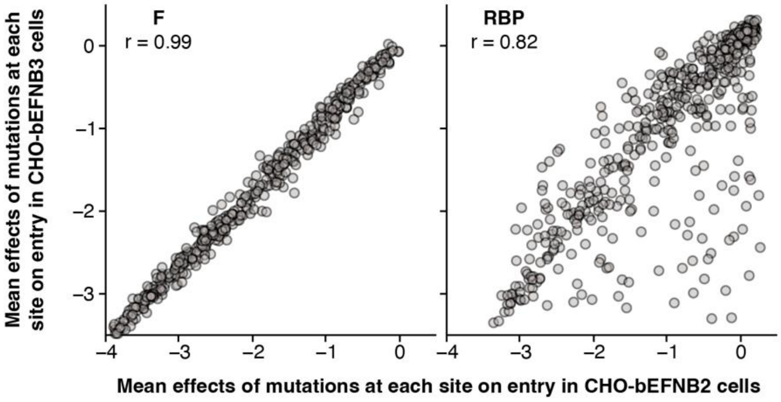
F
r = 0.99
RBP
r = 0.82
Mean effects of mutations at each site on entry in CHO-bEFNB3 cells
Mean effects of mutations at each site on entry in CHO-bEFNB2 cells

Fig. S6. Mean effects of F and RBP mutations at each site on cell entry in CHO-bEFNB2 or -bEFNB3 cells.
Correlation between the average effects of mutations on entry in CHO cells expressing either bEFNB2 or bEFNB3, with the measurements for F from the current study and RBP from Larsen et al. 2024 (2).

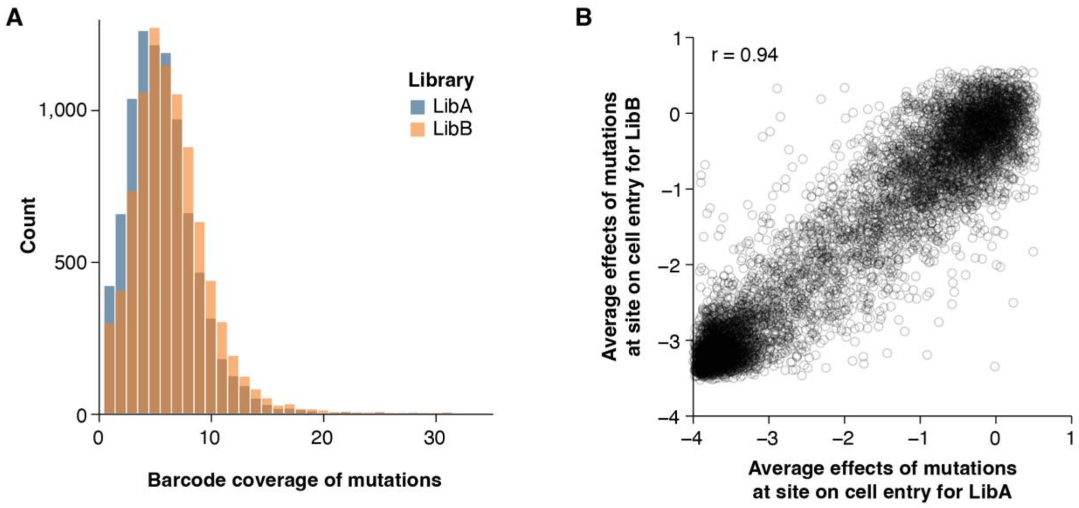

Fig. S7. Barcode coverage of variants and correlation between independent replicate libraries.
A) Number of unique barcodes covering each mutation following global epistasis fitting. Only mutations that were linked to at least two separate barcodes were included in the final analyses.
B) Correlation in cell entry between replicate libraries. Average effects of mutations on cell entry were calculated separately for four independent selections with either LibA or LibB. These averaged effects were then used to estimate the correlation between each independent library.

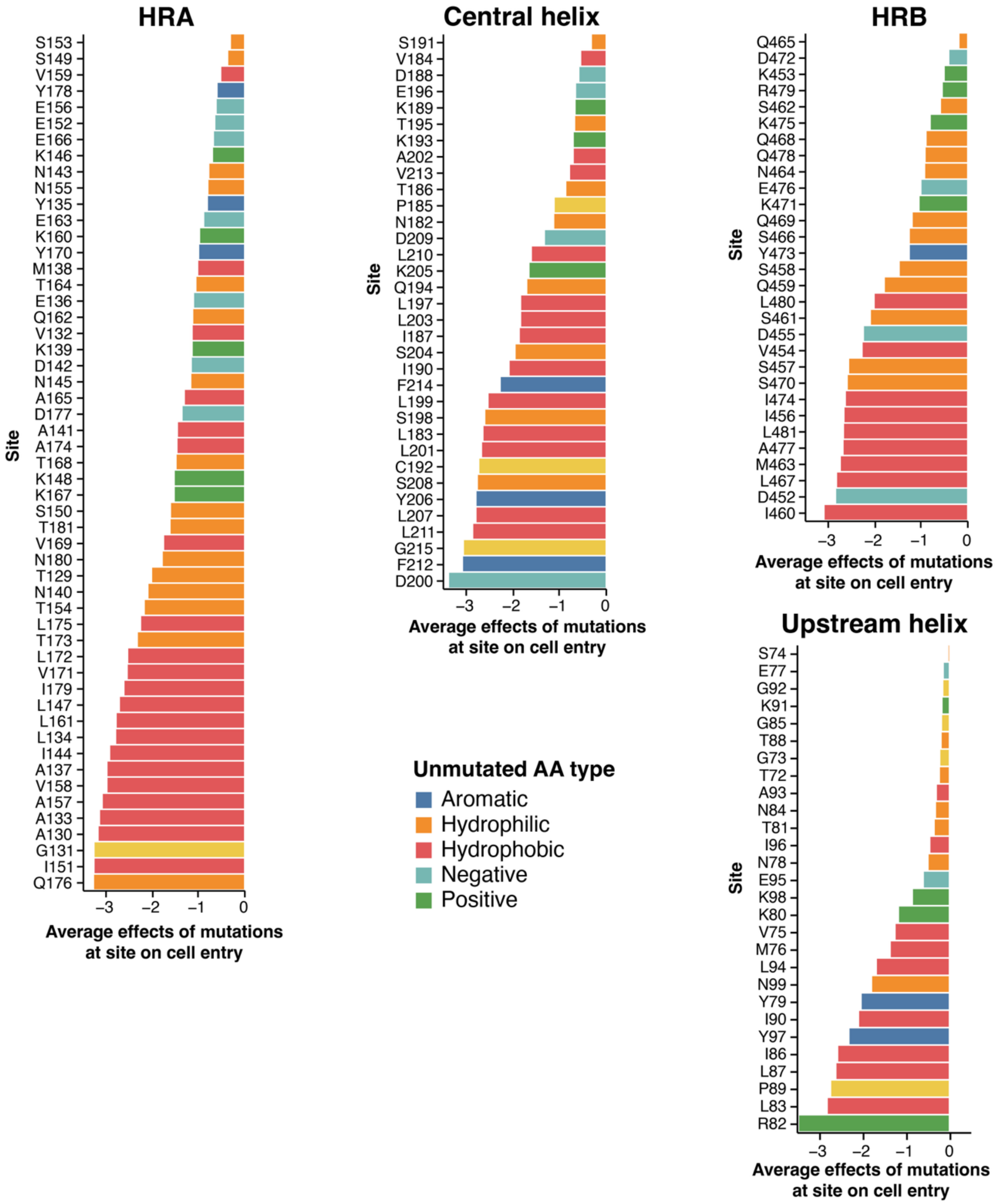

Fig. S8. Average effects of mutations at each site on cell entry in the heptad repeat regions.
Average effects of mutations at sites in the four main heptad repeat regions (Fig. 1). For each region, sites are ranked from least-constrained (top) to most-constrained (bottom) and are colored by the amino-acid property of the unmutated residue.

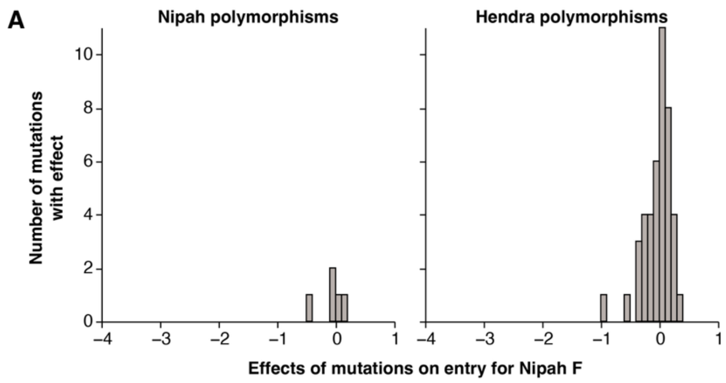

B

Site	Parental Nipah AA	Mutation	# Observed	Cell Entry
2	V	A	80	
6	D	N	80	
9	C	Y	80	
10	Y	N	2	
11	C	S	81	
13	L	F	2	
15	I	L	14	
19	M	I	21	
36	I	V	2	-0.4
42	V	I	81	0.0
249	A	T	2	0.0
273	S	G	79	0.1
367	L	M	3	-0.1
503	A	V	2	

C

Site	Parental Nipah AA	Mutation	# Observed	Cell Entry
42	V	I	16	0.0
68	M	V	20	-0.1
70	Q	K	20	0.1
74	S	T	20	-0.1
81	T	S	16	0.2
81	T	N	4	0.0
84	N	T	20	-0.2
88	T	S	20	0.2
94	L	I	20	0.1
96	I	L	20	-0.3
98	K	N	20	-0.6
105	V	I	4	-0.2
109	R	K	20	0.0
114	I	V	20	0.1
118	V	I	20	0.0
189	K	Q	20	0.2
198	S	A	19	-0.1
267	I	V	20	0.2
293	I	V	20	-0.3
316	I	V	20	-0.3
318	V	I	20	0.2
328	I	V	20	0.4
329	G	K	20	0.0
330	F	Y	20	-0.1
333	I	T	4	0.1
336	R	K	20	0.1
350	N	A	16	0.3
350	N	S	4	0.2
351	N	S	16	0.1
351	N	T	4	-0.9
361	E	D	20	-0.1
380	N	G	20	0.0
418	P	T	20	0.0
420	A	V	20	0.2
425	V	I	20	0.0
436	V	I	16	-0.4
436	V	T	4	-0.2
442	G	S	20	0.0
445	I	V	20	0.1
450	F	Y	20	0.0
453	K	R	4	0.2
479	R	K	16	-0.1
480	L	I	20	-0.3

Fig. S9. Effects of mutations on cell entry in circulating Nipah virus and Hendra virus F sequences.
A) Distribution of measured effects of mutations in the F ectodomain on cell entry for all publicly available Nipah and Hendra F sequences. Each unique mutation relative to the parental Nipah F sequence was counted once. Only mutations found in at least two sequences are included. **B)** Information about each mutation found in circulating sequences relative to the parental Nipah F sequence. Although there were 14 mutations that occurred at least twice, most occurred in the signal peptide, which was not mutagenized for our experiments. **C)** Hendra F mutations in

circulating sequences relative to the parental Nipah F sequence. Due to the large number of differences, only mutations that occurred in the ectodomain are shown.

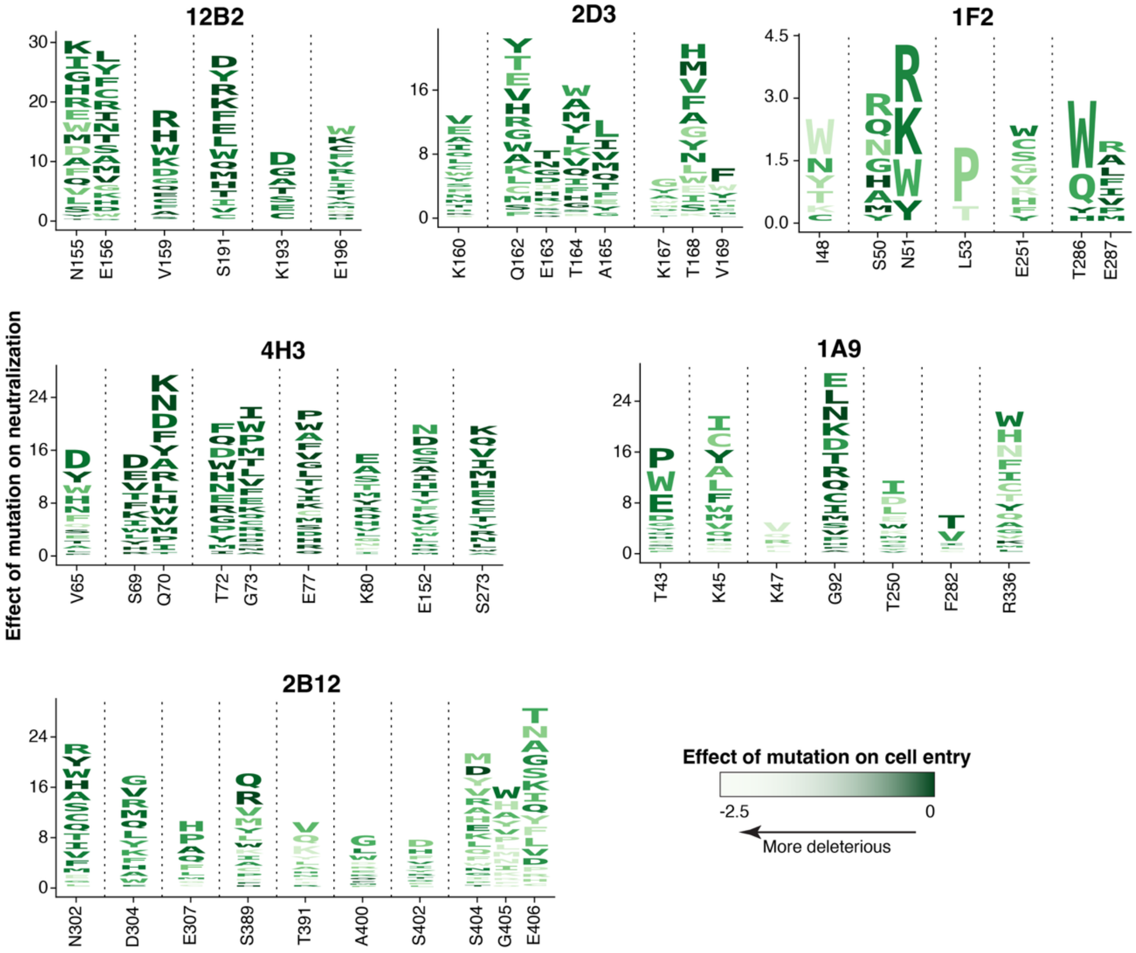

Fig. S10. Effects of F mutations on antibody neutralization and cell entry
Logo plots where the height of each letter is proportional to the amount a mutation decreases neutralization, and are colored by the effect that mutation has on cell entry, with darker colors indicating mutations that are neutral for cell entry, and lighter colors indicating mutations that are deleterious for cell entry. For each antibody, the logo plots show key sites where mutations have the greatest effect on neutralization.

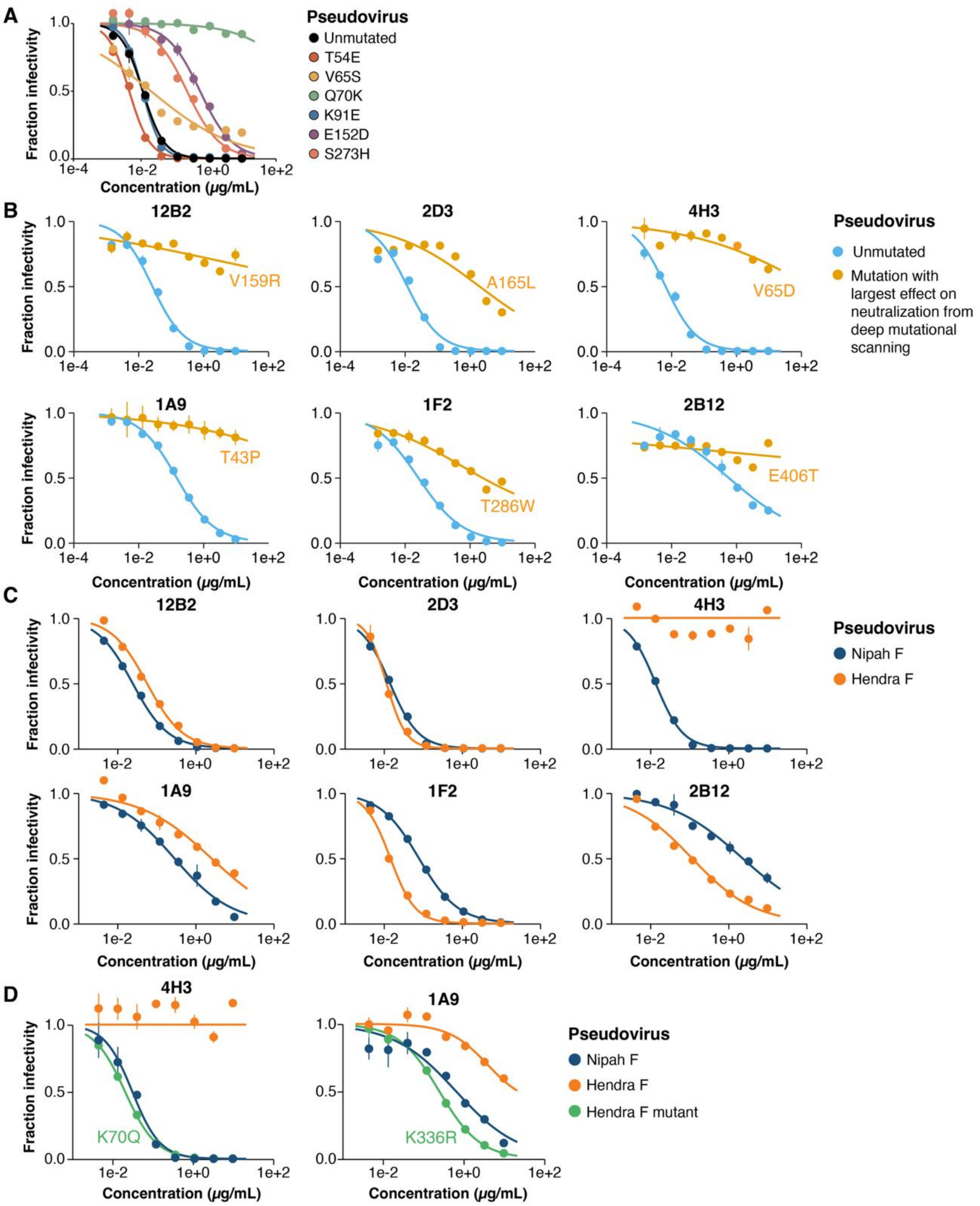

Fig. S11. Neutralization curves by each antibody towards pseudoviruses expressing unmutated Nipah F, single-mutants of Nipah or Hendra F, or unmutated Hendra F.
Raw neutralization curves used for estimating IC_{50} values for Figs. 6B, 6E, and 6G. Neutralization assays were performed using luciferase-based lentiviral particles pseudotyped with different Nipah F single mutations or Hendra F. Experiments were performed in duplicate. **A)** Neutralization curves of unmutated Nipah F and six different single mutations with the antibody 4H3. Individual mutations were selected to span a range of effects on neutralization. **B)** Neutralization curves of unmutated Nipah F and the mutation with the largest effect on neutralization for each antibody. **C)** Neutralization curves of unmutated Nipah and Hendra F. **D)**

Neutralization curves of pseudoviruses expressing the unmutated Nipah receptor binding protein and either unmutated Hendra F, unmutated Nipah F, or Hendra F with specific mutations that revert the Hendra F to the amino-acid identity in the Nipah F at a site where this is expected to confer sensitivity to antibody neutralization.

SI References

1. K. M. Esvelt, Inoculating science against potential pandemics and information hazards. *PLoS Pathog.* **14**, e1007286 (2018).
2. G. Lewis, P. Millett, A. Sandberg, A. Snyder-Beattie, G. Gronvall, Information Hazards in Biotechnology. *Risk Anal.* **39**, 975–981 (2019).
3. B. B. Larsen, *et al.*, Functional and antigenic landscape of the Nipah virus receptor-binding protein. *Cell* **188**, 2480–2494.e22 (2025).
4. P. O. Byrne, *et al.*, Structural basis for antibody recognition of vulnerable epitopes on Nipah virus F protein. *Nat. Commun.* **14**, 1494 (2023).
5. H. V. Dang, *et al.*, Broadly neutralizing antibody cocktails targeting Nipah virus and Hendra virus fusion glycoproteins. *Nat. Struct. Mol. Biol.* **28**, 426–434 (2021).
6. B. Dadonaite, *et al.*, A pseudovirus system enables deep mutational scanning of the full SARS-CoV-2 spike. *Cell* **186**, 1263–1278.e20 (2023).
7. M. Weis, A. Maisner, Nipah virus fusion protein: Importance of the cytoplasmic tail for endosomal trafficking and bioactivity. *Eur. J. Cell Biol.* **94**, 316–322 (2015).
8. L. E. MacConaill, *et al.*, Unique, dual-indexed sequencing adapters with UMIs effectively eliminate index cross-talk and significantly improve sensitivity of massively parallel sequencing. *BMC Genomics* **19**, 30 (2018).
9. K. H. D. Crawford, J. D. Bloom, alignparse: A Python package for parsing complex features from high-throughput long-read sequencing. *J Open Source Softw* **4** (2019).
10. J. Otwinowski, D. M. McCandlish, J. B. Plotkin, Inferring the shape of global epistasis. *Proc. Natl. Acad. Sci. U. S. A.* **115**, E7550–E7558 (2018).
11. H. K. Haddox, *et al.*, Jointly modeling deep mutational scans identifies shifted mutational effects among SARS-CoV-2 spike homologs. *bioRxivorg* 2023.07.31.551037 (2023).
12. T. C. Yu, *et al.*, A biophysical model of viral escape from polyclonal antibodies. *Virus Evol* **8**, veac110 (2022).
13. K. Katoh, D. M. Standley, MAFFT multiple sequence alignment software version 7: improvements in performance and usability. *Mol. Biol. Evol.* **30**, 772–780 (2013).
14. B. Q. Minh, *et al.*, IQ-TREE 2: New Models and Efficient Methods for Phylogenetic Inference in the Genomic Era. *Mol. Biol. Evol.* **37**, 1530–1534 (2020).
15. E. C. Meng, *et al.*, UCSF ChimeraX: Tools for structure building and analysis. *Protein Sci.* **32**, e4792 (2023).
16. J. Jumper, *et al.*, Highly accurate protein structure prediction with AlphaFold. *Nature* **596**, 583–589 (2021).
17. M. Mirdita, *et al.*, ColabFold: making protein folding accessible to all. *Nat. Methods* **19**, 679–682 (2022).
18. Z. Wang, *et al.*, Structure and design of Langya virus glycoprotein antigens. *Proc. Natl. Acad. Sci. U. S. A.* **121**, e2314990121 (2024).
19. D. S. Zyla, *et al.*, A neutralizing antibody prevents postfusion transition of measles virus fusion protein. *Science* **384**, eadm8693 (2024).
20. R. J. Loomis, *et al.*, Structure-Based Design of Nipah Virus Vaccines: A Generalizable Approach to Paramyxovirus Immunogen Development. *Front. Immunol.* **11**, 842 (2020).
21. P. O. Byrne, *et al.*, Prefusion stabilization of the Hendra and Langya virus F proteins. *J. Virol.* **98**, e0137223 (2024).
